# Comprehensive analysis of differentially expressed rice actin depolymerizing factor gene family and heterologous overexpression of *OsADF3* confers *Arabidopsis Thaliana* drought tolerance

**DOI:** 10.1186/1939-8433-5-33

**Published:** 2012-11-27

**Authors:** Ya-Chen Huang, Wen-Lii Huang, Chwan-Yang Hong, Hur-Shen Lur, Men-Chi Chang

**Affiliations:** Department of Agronomy, National Taiwan University, No. 1, Section 4, Roosevelt Road, Taipei, 106 Taiwan Republic of China; Department of Agronomy, National Chiayi University, No.300 Syuefu Rd., Chiayi, 60004 Taiwan Republic of China; Department of Agriculture Chemistry, National Taiwan University, No. 1, Section 4, Roosevelt Road, Taipei, 106 Taiwan Republic of China

**Keywords:** Rice (*Oryza sativa* L.), Actin depolymerizing factor, Gene expression profiling, Particle bombardment, GUS staining, Heterologus overexpression, Abiotic stresses

## Abstract

**Background:**

Actin depolymerizing factors (ADFs) are small actin-binding proteins. Many higher-plant ADFs has been known to involve in plant growth, development and pathogen defense. However, in rice the temporal and spatial expression of OsADF gene family and their relationship with abiotic stresses tolerance is still unknown.

**Results:**

Here we reported the first comprehensive gene expression profile analysis of *OsADF* gene family. The *OsADF* genes showed distinct and overlapping gene expression patterns at different growth stages, tissues and abiotic stresses. We also demonstrated that both OsADF1 and OsADF3 proteins were localized in the nucleus. *OsADF1* and *OsADF3* were preferentially expressed in vascular tissues. Under ABA or abiotic stress treatments, *OsADF3*::*GUS* activity was enhanced in lateral roots and root tips. Ectopically overexpressed *OsADF3* conferred the mannitol- and drought-stress tolerance of transgenic *Arabidopsis* seedlings by increasing germination rate, primary root length and survival. Several drought-tolerance responsive genes (*RD22*, *ABF4*, *DREB2A*, *RD29A*, *PIP1*; *4* and *PIP2*; *6*) were upregulated in transgenic *Arabidopsis* under drought stress.

**Conclusions:**

These results suggested that OsADF gene family may participate in plant abiotic stresses response or tolerance and would facilitate functional validation of other *OsADF* genes.

**Electronic supplementary material:**

The online version of this article (doi:10.1186/1939-8433-5-33) contains supplementary material, which is available to authorized users.

## Background

The plant actin cytoskeleton is involved in a range of cellular processes, including stress response (reviewed in Hussey et al., 2006; Staiger and Blanchoin, 2006; Drobak et al., 2004). Intracellular actin filament activity is modulated by a number of actin binding proteins such as profillin, actin depolymerizing factor (ADF)/cofilin, myosin, fibrin and villin. Plant ADFs with low molecular weight (16–20 kD) can act synergistically with profillin to increase the turnover rates and sever actin filaments (Staiger et al., 1997). The interaction between actin and ADF is regulated by reversible phosphorylation, pH, and specific phosphoinositides (Allwood et al., 2002; Smertenko et al., 1998).

The temporal and spatial expression of higher-plant ADFs has gradually been deciphered, but not with rice OsADF gene family. In Arabidopsis, ADF gene expression can be separated into vegetative- and reproductive-specific classes (Ruzicka et al., 2007). In cotton, *GhADF6* and *GhADF8* express mainly in petals, whereas *GhADF7* expression is anther specific (Li et al., 2010). In lily and maize, *LiADF1* and *ZmADF1*/*2* accumulate solely in pollen, whereas *ZmADF3* is expressed differentially in vegetative tissues (Jiang et al. 1997). The subcellular localization of various AtADFs was intensively studied by histochemical staining of *AtADF*::*GUS* fusion genes. Two classes of AtADFs may co-evolve in a tissue and developmental-specific manner and mediate distinct functions (Ruzicka et al., 2007). As well, intron-mediated enhancement of ADF gene expression was reported in vascular bundle tissue of *Arabidopsis* (*AtADF1*) and petunia (*PhADF1*) (Mun et al., 2002; Jeong et al., 2009).

Little is known about the precise physiological function and role of members of the plant ADF gene family. Specific members are important for plant growth, development and viability. ADFs are involved in pollen tube growth with dynamic cytoskeleton rearrangement (Allwood et al., 2002; Lopez et al., 1996). The moss *Physcomitrella patens* contains only a single essential ADF gene, and loss of *PpADF* led to inhibited tip growth (Augustine et al., 2008). In *Arabidopsis*, the *AtADF9* mutant, which is moderately expressed in the shoot apical meristem, shows few lateral branches, reduced callus formation, early flowering, associated with less active chromatin state of F lowering L ocus C (Burgos-Rivera et al., 2008). The downregulation of *GhADF1* expression affected cotton fiber properties by increasing fiber length and strength (Wang et al., 2009).

Recently, plant actin cytoskeleton had been shown to play an important role in response to plant hormones and biotic or abiotic stresses (Solanke and Sharma, 2008; Drobak et al., 2004). ADFs from *Arabidopsis* (AtADF2 and AtADF4) and barley were found related to plant resistance to various pathogens (Clement et al., 2009; Miklis et al. 2007; Tian et al., 2009). Alteration in the core amino acid residue in moss ADF (ADF-V69A) allowed the plant to grow at a permissive temperature (20°C to 25°C) but not a restrictive temperature (32°C; Vidali et al., 2009). Heat stress induced depolymerization of actin microfilaments and changed endoplasmic reticulum morphologic features in tobacco BY2 cultured cells (Malerba et al., 2010). Moreover, in winter oilseed rape suspension cells, freezing-induced depolymerization of actin microfilaments was sensitive in the cell growth phase (Egierszdorff and Kacperska, 2001). During cold acclimation, *TaADF* accumulated to higher levels in freezing-tolerant but not -sensitive wheat cultivars. This ADF was specifically induced by low temperature but not salt or heat (Ouellet et al., 2001). However, Basisakh and Subudhi (2009) identified an ADF gene in smooth cordgrass (*Spartina alterniflora* L.) that was highly induced with salt and heat stress in leaf and shoot but only heat stress in root. Proteomic analysis revealed induction of OsADF in vegetative-stage rice leaves of an upland cultivar CT9993 under drought stress that disappeared on re-watering but remained unaffected in the lowland cultivar IR62266 (Salekdeh et al. [Bibr CR40][Bibr CR41]). The expression of OsADF3 (GeneBank: AC104433) was induced by drought and osmotic stresses but not salt, cold or ABA in the leaf sheath of rice seedlings (*Oryza sativa* L. cvs. Nipponbare and Zhonghua 8) (Ali and Komatsu, 2006). However, OsADF3 protein was induced by salt stress in Nipponbare root (Yan et al., 2005). OsADF3 protein could be induced by exogenous ABA and may be involved in altering the morphologic features of Taichung native 1 (TCN1) rice root growth and development (Chen et al., 2006). Interestingly, cDNA-amplified fragment length polymorphism analysis revealed induced expression of *OsADF2* (GeneBank: AC084320) under drought stress in the seminal root of the upland rice cultivar Azucena (Yang et al., 2003).

Investigating abiotic stress associated OsADF expression is important to determine whether the genes are involved in abiotic stress tolerance in rice. Nevertheless, a comprehensive analysis of gene expression patterns of the rice ADF gene family has not been performed yet. To reveal the physiological role of *OsADFs*, we characterized the temporal and spatial gene expression patterns of the *OsADF* gene family in different tissues, growth stages and under various abiotic stresses of rice. We determined the subcellular localization and promoter activity of OsADF1 and OsADF3 genes. We also overexpressed *OsADF3* in *Arabidopsis* to provide further evidence of the *OsADF3* function in enhancing drought/osmotic stress tolerance of transgenic *Arabidopsis* by modulating several downstream abiotic stress-responsive target genes related to drought responses.

## Results

### Expression profile analysis of *OsADFs* in different tissues, developmental stages under ABA or abiotic stress

To understand the tissue, developmental specificity and abiotic stress responses of the expression of *OsADF* genes, we manually re-annotated OsADF gene structures (Additional file 1: Table S2) and performed 5’-RACE to determine the corresponding 5’ transcription initiation sites (data not shown). Then, we performed phylogenetic analysis of ADFs from *Arabidopsis* and rice (Additional file 2: Figure S1 and Additional file 3: Figure S2). Finally, we analyzed the abiotic stress-related *cis*-acting elements, including ABA-responsive element (ABRE), dehydration-responsive element/C-repeat (DRE/CRT) and low-temperature responsive element (LTRE), in the 1-kb promoter regions of *OsADF* promoters (Additional file 4: Figure S3). In addition, we investigated rice microarray data downloaded from GEO (Accession No. GSE6901 and GSE6893) to gain insight into the transcript levels of different members of *OsADFs* in various tissues and abiotic stresses. The expression of *OsADF3* was induced by salt and drought, and that of *OsADF5* was less induced. In contrast, the expression of *OsADF7* and *OsADF11* was slightly reduced with salt and drought. The expression of *OsADF2* and *OsADF4* was not changed with stress. Most genes, such as *OsADF1*, *3*, *5*, *6*, *7* and *9*, seemed to preferentially express in rice spikelets. The expression of *OsADF11* was increased in leaf tissue (data not shown).

**Figure 1:**
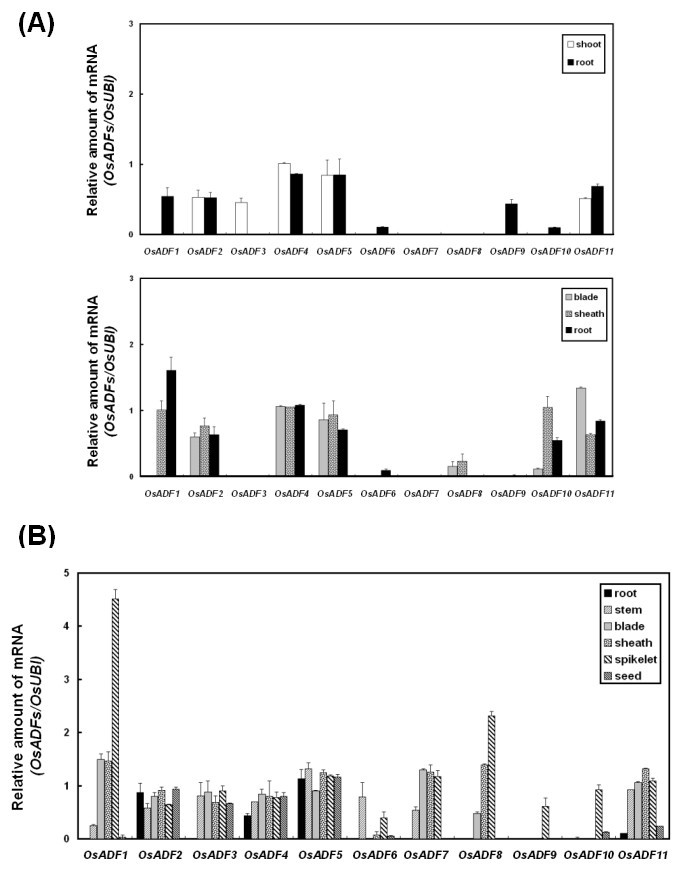
**Expression patterns of rice actin depolymerizing factors (OsADFs) in different tissues of rice at various developmental stages from 12-, 45- to 90-day-old.** Transcript levels of OsADFs were determined in shoot and root of 12-day-old rice seedlings (upper) and in leaf blade, leaf sheath and root at early tillering stage (45 days old) (lower) **(A)**. Transcript levels of OsADFs were determined in root, stem, leaf blade, leaf sheath, spikelet and seed at heading stage (90 days old) **(B)**. OsADF expression is relative to that of the rice ubiquitin gene OsUBI (D12629) used as an internal control. Bars show means ± SE (n = 3).

We took above information for RT-PCR analysis to determine the transcript levels of individual members of *OsADF* genes. As predicted from the microarray dataset, all OsADF genes were predominantly expressed in rice spikelets, with the expression of *OsADF1* the highest (Figure 1A and B, Additional file 5: Figure S4). *OsADF9* showed a unique spikelet-specific gene expression. However, *OsADF2*, *4*, *5* and *11* were expressed in all tissues examined. *OsADF3*, *7* and *11* expressed mainly in stem, leaf blade, sheath and spikelet, as did *OsADF8* but not in stem. *OsADF6* transcripts were only in stem and spikelets, whereas those of *OsADF10* were also in rice spikelets but relatively low in seeds. *OsADF2*, *4*, *5* and *11* persistently expressed in different growth stages of rice (Figure 1A and B, Additional file 5: Figure S4). *OsADF1* and *10* expressed only in leaf sheath and root during early tillering stage, whereas *OsADF6* and *9* expressed predominantly in roots at seedling and early tillering stages. The mRNA level of *OsADF1* was relatively high in leaf sheaths and root of early tillering whereas that of *OsADF3* was only slightly detected in seedling shoot.

**Figure 2:**
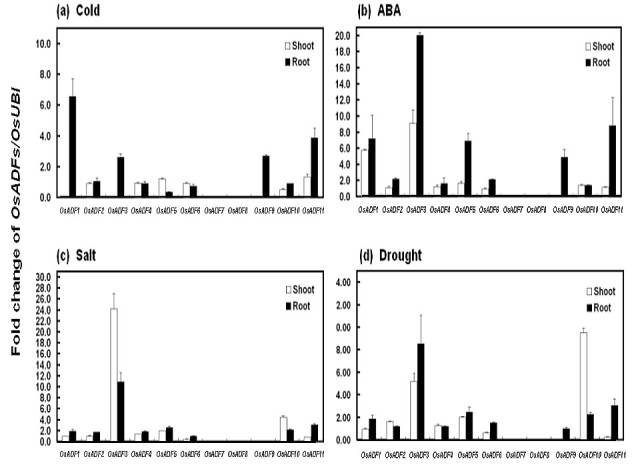
**Expression patterns of** ***OsADFs*** **under various abiotic stresses and abscissic acid (ABA) treatment in root or shoot of 12-day-old rice seedlings.** OsADF expression is relative to that of OsUBI (D12629) used as an internal control. After adjustment, the control values of *OsADF* mRNA expression in shoot or root were arbitrarily assigned a value of 1 as a normalized reference for determining the relative mRNA amount of other *OsADF* genes. Bars show means ± SE (n = 3).

No gene was induced by cold in the shoot (Figure 2 and Additional file 6: Figure S5). *OsADF1* and *3* were induced by ABA; *OsADF1*, *3*, *4*, *5*, *10* and *11* were induced by salt; and *OsADF3*, *5* and *10* were induced by drought. In root, *OsADF1*, *3*, *9*, and *11* were induced by cold; *OsADF1*, *3*, *5*, *9* and *11* were induced by ABA; *OsADF3*, *5*, *6*, *10* and *11* were induced by drought; and *OsADF3* was induced by salt. Surprisingly, the mRNA expression of *OsADF9* in root was repressed by salinity stress.

### Subcellular localization of OsADF1 and OsADF3 proteins in onion epidermal cells

**Figure 3:**
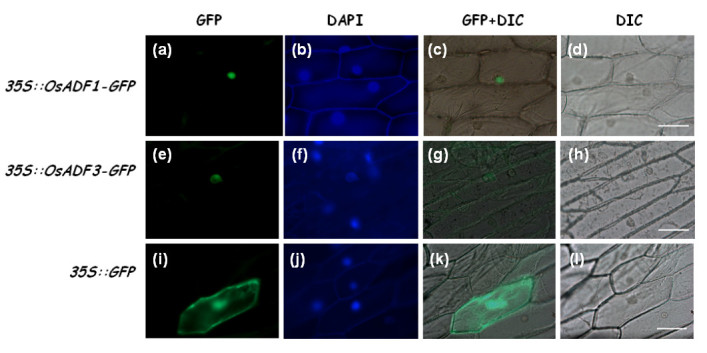
**Localization of OsADF1-GFP and OsADF3-GFP fusion proteins.** Onion epidermal cells were bombarded with plasmids as indicated; fluorescence microscopy of p*ubi*::OsADF1-GFP **(a**-**d)**; p*ubi*::OsADF3-GFP **(e**-**h)** and p*ubi*::GFP **(i**-**l)**. GFP fluorescence **(a**, **e**, **i)**; DAPI staining **(b**, **f**, **j)**; merged GFP/bright-field (DIC; **c**, **g**, **k**) and bright-field **(d**, **h**, **l)**. Bars = 100 μm.

Several proteomics studies suggest that OsADF1 and 3 express under drought stress and may function in tolerance to drought stress, especially in the upland rice variety (Yan et al., 2005; Chen et al., 2006). However, little is known regards to the subcellular localization of protein expression. Thus, we chose these two OsADF gene members as initial candidates for protein localization and promoter activity assay. The N terminus of OsADF1 and OsADF3 contains a non-typical nuclear localization signal (NLS) amino acid sequence (KRXHP) (Maciver and Hussey, 2002) (Additional file 2: Figure S1). The OsADF1-GFP and OsADF3-GFP constructs were introduced into onion epidermal cells by microprojectile bombardment. The result indicated that OsADF1 (Figure 3a–d) and OsADF3 (Figure 3e–h) were in the nucleus while the control GFP protein distributed across the cytoplasm and nucleus of onion epidermal cells (Figure 3i–l).

### Histochemical analysis of GUS expression patterns in *p*_*OsADF1i*_::*GUS* and *p*_*OsADF3i*_::*GUS* transgenic rice

**Figure 4:**
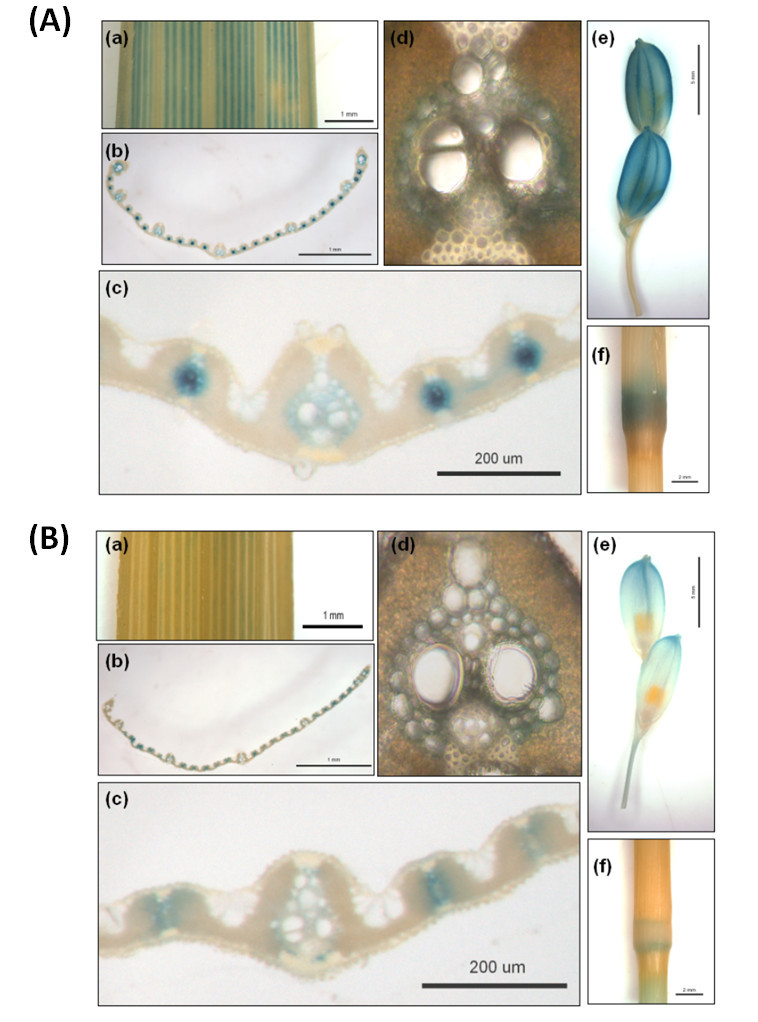
**GUS expression in leaf, spikelet and internode of** ***p***_***OsADF1i***_**::** ***GUS*** **(A) and** ***p***_***OsADF3i***_**::** ***GUS*** **(B) transgenic rice (T**_**0**_**) plants.** GUS staining in **(a)** mature leaf; **(b**-**d)** different magnified views of leaf blade cross-sections; and **(e)** spikelets and **(f)** internode, respectively.

**Figure 5:**
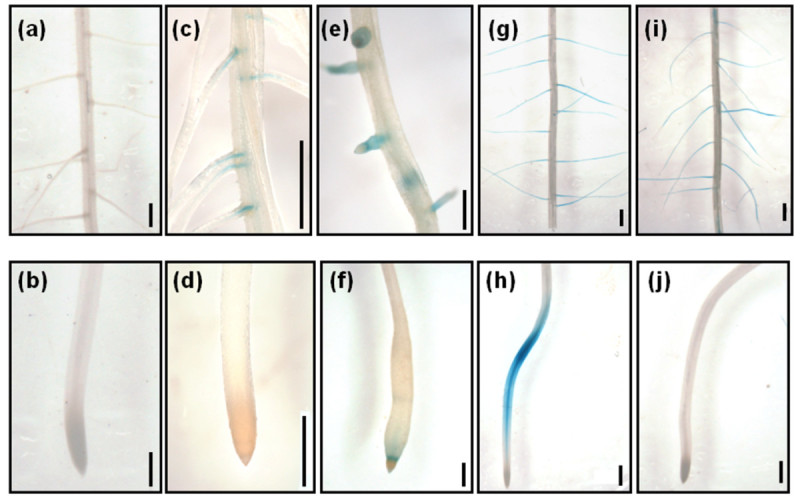
**Effect of various abiotic stresses or ABA treatment on GUS expression in vascular tissue of primary or lateral root regions of** ***p***_***OsADF3i***_**::** ***GUS*** **transgenic rice (T**_**1**_**) plants.** The top panels **(a**, **c**, **e**, **g**, and **i)** represents the images taken from lateral roots and the bottom panels **(b**, **d**, **f**, **h**, and **j)** are from primary root tips. GUS staining of **(a**, **b)** roots under normal growth conditions; **(c**, **d)** roots after 24-hr cold treatment; **(e**, **f)** roots after 10-μM ABA treatment for 24 hr; **(g**, **h)** roots after 200 mM salt solution treatment for 24 hr; and **(i**, **j)** roots after air-dried treatment for 2.5 hr. Bars = 1 mm.

Intron-dependent spatial and enhanced gene expression in *Arabidopsis ADFs* was reported (Jeong et al., 2009). Thus, we analyzed the promoter expression activities of *OsADF1* and *OsADF3* with the GUS constructs containing the first intron (*pOsADF1i* or *pOsADF3i*) in transgenic rice. The GUS expression pattern of *OsADF1* and *OsADF3* were mainly detected in rice vascular tissue, such as leaf blade (Figure 4A, B (a-d)), the nodule of internodes (Figure 4A, B (f)) and veins in the seed husk (Figure 4A, B (e)). Interestingly, after cold, salt, ABA, salt and air-dried treatments, lateral roots and root tips of transgenic rice seedlings showed a distinct pattern of *OsADF3* promoter-driven GUS expression. Transgenic rice seedling roots with no treatment showed no GUS signal (Figure 5(a), (b)). However, GUS staining was especially observed in the emergence of lateral roots under low temperature (Figure 5(c), (d)). The lateral root primordial and root tips showed strong GUS expression with ABA treatment (Figure 5(e), (f)). Under salt and air-dried conditions, the lateral roots of transgenic rice seedling all showed GUS staining (Figure 5(g), (i)), which was extended along the primary root with salt treatment (Figure 5(h), (j)).

### Phenotypic analysis of transgenic *OsADF3*(*OsADF3*-*OE*) *Arabidopsis* plants under mannitol or drought stress

**Figure 6:**
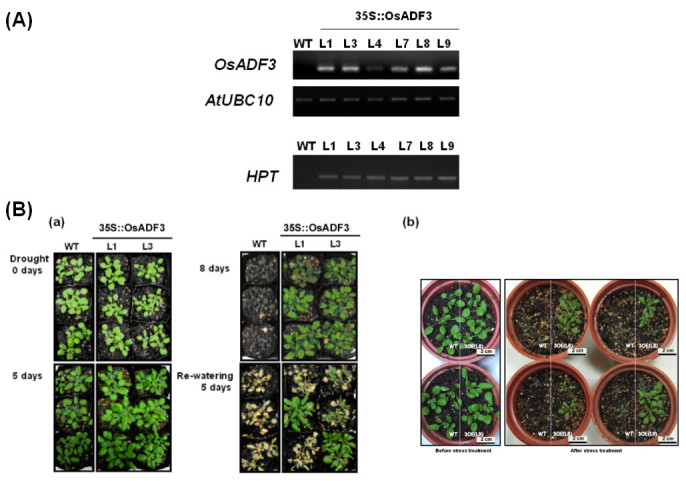
**Molecular characterization and effect of drought stress on soil-grown** ***p35S*** **::** ***OsADF3*** **transgenic** ***Arabidopsis*** **plants.** **(A)** RT-PCR analysis of *OsAD3* mRNA expression in transgenic and wild-type plants. The *HPT* (hygromycin) marker was used for selection of homozygous plants. **(B)** For drought-stress tolerance assay, 3-week-old wild type and transgenic plants were grown in standard soil condition, then water was withheld for 12 days and plants were re-watered for 5 days. Photographs correspond to plants at day 5 and 8 of water deficit (**(a)**, transgenic lines L1 and L3), recovery of water stress treatment for 5 days (**(a)** and **(b)**, lines L1, L3, L8 and L9).

**Figure 7:**
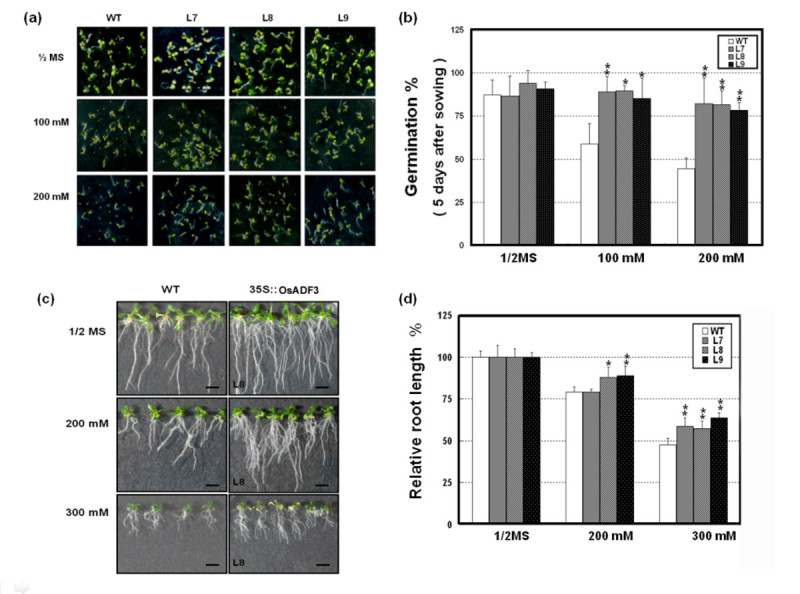
**Effect of mannitol on** ***OsADF3*** **overexpressed (** ***OsADF3*** **-** ***OE*** **)** ***Arabidopsis*** **plants.** **(a)** Seeds were sown in triplicate on standard medium with different concentrations of mannitol (0–300 mM) for continuous growth for 5 days. **(b)** Radicle emergence was recorded and data are means ± SD (n = 30–35 plants) **(c)** One-week-old seedlings grown on standard medium were transferred to culture plates with concentrations of mannitol (0–300 mM) for 7 days. **(d)** The primary root length was measured by use of Image J and data are means ± SD (n = 6). *P < 0.05; **P < 0.01 compared with the wild type.

To examine the functions of *OsADF3*, we analyzed the effect of *OsADF3* ectopically overexpressed in *Arabidopsis*. We obtained more than 10 T4-transgenic plants and homozygous plants were characterized by genomic PCR genotyping with the *HPT* marker (Figure 6A). Transgenic lines with the relatively highest *OsADF3* levels (L1, 3, 8 and 9) were used for phenotype analysis (Figure 6A). Under normal conditions, growth of *OsADF3*-*OE* and wild-type (WT) *Arabidopsis* did not differ. After exposure to water deficit for 5 days, WT plants accumulated a high amount of anthocyanin and showed severely decreased leaf areas and reduced biomass. However, *OsADF3*-*OE* plants still showed no phenotype difference (Figure 6B (a)). When plants recovered from 12 days of water deprivation with re-watering for 5 days, WT plants were dead with chlorotic leaves, whereas most transgenic *Arabidopsis* continued to grow, albeit at a slower rate (Figure 6B (a) and (b)). We further investigated the effect of mannitol on seed germination and inhibition of primary root elongation in *OsADF3*-*OE Arabidopsis* lines L7, L8 and L9 and the WT. Without mannitol treatment, WT and transgenic lines showed a similar germination rate. In contrast, with mannitol concentration > 100 mM, the germination rate of WT seeds was decreased to 50% (at 200 mM mannitol), whereas *OsADF3*-*OE Arabidopsis* still maintain their germination capacity (Figure 7(a) and (b)). In the absence of mannitol, WT and *OsADF3*-*OE* transgenic plants did not differ in root growth (Figure 7(c) and (d)); however, at 300 mM mannitol, the primary root growth was inhibited to about 40% in transgenic lines and to about 60% in WT plants.

### Expression of downstream abiotic stress-responsive target genes in transgenic *OsADF3*-*OE Arabidopsis* plants

**Figure 8:**
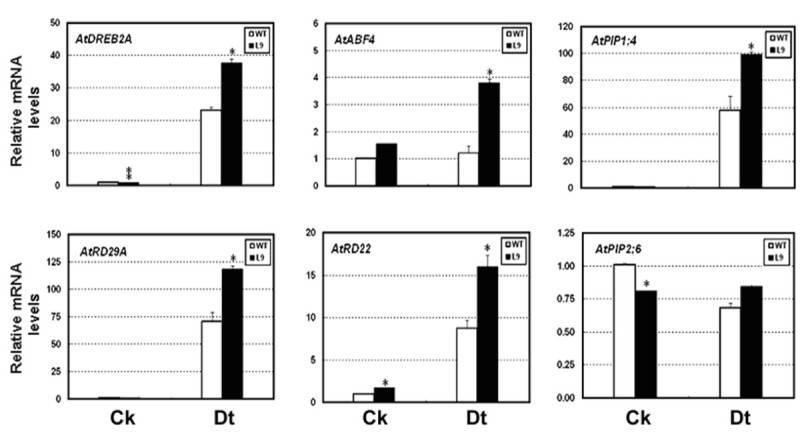
**Quantitative RT-PCR analysis of drought-stress–responsive mRNA expression in wild type and transgenic** ***Arabidopsis*** **(L9).** Fourteen-day-old seedlings were not treated (C_K_) or air-dried for 2.5 hr (D_t_) and the relative mRNA levels of drought-tolerance–related genes in wild-type (white bars) and *OsADF3*-*OE Arabidopsis* line L9 (black bars) were analyzed by real-time PCR. *AtUBC10* (At5G53300) was an internal control. Data are representative of 3 independent experiments. *P < 0.05; **P < 0.01 compared with the wild type.

Several drought-stress induced genes, including aquaporins (plasma membrane intrinsic proteins (*PIPs*)), *RD22*, *RD29B* and *RD29A* were known to confer dehydration stress tolerance in *Arabidopsis* (review by Shinozaki and Yamaguchi-Shinozaki, 2007). With dehydration stress, the gene expression of *AtPIP1*;*4* was strongly induced, whereas that of *AtPIP2*;*6* was downregulated (Alexandersson et al., 2010). Other gene such as *DREB2A* can bind to the dehydration responsive element (DRE) in the promoter of *RD29A* (Narusaka et al., 2003; Sakumaa et al. 2006). ABF4 and MYB2 mediate the upregulation of *RD22* and *RD29B* gene expression during water deficit. (Kang et al., 2002; Abe et al., 2003). To understand the increase in mannitol- and drought-stress tolerance of transgenic *OsADF3OE Arabidopsis*, we used real-time PCR analysis to verify the expression of these genes. With air-dried treatment, the expression of *DREB2A* and *RD29A* was significantly induced (Figure 8). ABA up-regulated gene expression of *ABF4*, *RD22* and *AtPIP1*;*4* was also increased in transgenic lines. The expression of *AtPIP2*;*6* was down regulated in *OE* lines under normal conditions but maintained at levels similar to the WT under drought stress. *MYB2* and *RD29B* showed inconsistent change in gene expression (data not shown).

## Discussion

### The monocots and dicots ADF gene family may not evolve with the same function and display similar gene expression patterns

To understand the diverse function of each *OsADF* member, *OsADF* and *AtADF* genes were categorized into 6 clades (A-F) according to the protein sequence alignment comparison and un-rooted phylogenetic tree analysis (Additional file 2: Figure S1 and Additional file 3: Figure S2). In clades A, B and C, *OsADFs* and *AtADFs* were both present and may origin from a common ancestor. Clade D was dicot specific and includes *AtADF1*, *2*, *3* and *4* that strongly expressed in vegetative and reproductive tissues, excluding pollen (Ruzicka et al., 2007). On the contrary, clades E and F are monocot specific and interestingly, *OsADF7* was distinguished from the others by its extraordinary length. The comparison of gene expression profiles between OsADFs and AtADFs gene family showed that *OsADF2* and *11* (clade A) similar to *AtADF6* were expressed in all tissues at a moderate level (Figure 1). In clade C, *OsADF1*, *6 and 9* all showed spikelet-preferential or specific gene expression, with *OsADF1* having the highest expression (Figure 1). This finding is consistent with AtADF *7* and *10* that predominately expressing in mature pollen or pollen tubes (Ruzicka et al., 2007). However, in clade B *OsADF5* was strongly and constitutively expressed in all tissues (Figure 1), but not *AtADF5* and *9*, which expressed weakly in vegetative stages except differentiating cells. *OsADF7* was previously grouped into clade I (Ruzicka et al., 2007) and Bi (Feng et al., 2006) showed a preferential expression at seedling but not early tillering stages (Figure 1). The OsADF genes within monocot-specific clades E and F share low amino acid sequence similarity (39–52%) and except for *OsADF4*, express constitutively in various tissues and developmental stages, with varied expression of other genes. The highly associated expression patterns in clades A and C may be a good indicator of a conserved function of related ADF proteins in *Arabidopsis* and rice. However, the diversification of monocot-specific clades and distinct gene expression profiles indicate that *Arabidopsis* and rice ADF genes may not evolve with the same function.

### OsADF1 and OsADF3 were nucleus localized and preferentially expressed in vascular tissues

Previously, immunocytochemical analysis of *Arabidopsis* revealed subclass I AtADFs localized in both cytoplasm and nucleus but subclass II genes localized in the cytoplasm only (Ruzicka et al., 2007). Surprisingly, we detected OsADF1 and OsADF3 proteins only in the nucleus in onion epidermal cells, despite non-classical nuclear localization signals in the N terminus (Figure 3). In *Dictyostelium* and *Z*. *mays*, ADF/cofilin proteins, which lack the classical bipartite nuclear localization signal, could enter the nucleus after 10% DMSO or cytochalasin D treatment (Maciver and Hussey, 2002). Whether both di- and monocot plant ADFs can behave like animal ones as stimulus-responsive modulators to cause actin cytoskeleton remodeling remains unknown (Nick, 2008).

In addition to gene expression profiling characterization (Figure 1 and Figure 2) and subcellular localization of gene-encoded protein (Figure 3), promoter activity assay also provide important information for revealing putative biological gene function. The promoter activities of *OsADF1* and *OsADF3* were all highly accumulated in vascular tissue–preferential vegetative organs in rice (Figure 4). A similar GUS expression pattern of petunia *phADF1* promoter was reported (Mun et al., 2002). ADF should coordinate with ubiquitinously expressed actin to cause cytoskeleton remodeling to alter cell shape or cell wall reorganization, growth or other physiological responses. Recently, Lefebvre et al., 2011 showed an *Arabidopsis* mutant (*esk1*) that decreased in cold, salt tolerance and water use efficiency was severely defective in chemical composition of xylem cell wall, altered vascular tissues and impaired water transport. It would be interesting to further determine why *OsADF1* and *OsADF3* tend to be bundle-preferential accumulated and how OsADF1 and OsADF3 may interact with vascular-specific actin or profiling.

### OsADF3 may enhance drought stress tolerance in plant through the activation of downstream abiotic stress-responsive target genes

To test OsADF3 gene function, we heterologuslly overexpressed *OsADF3* in *Arabidopsis* and found that the increase of drought stress tolerance and downstream drought-tolerant responsive genes expression (Figures 6, 7 and 8). Actin was traditionally considered an abundant cytoskeleton protein with numerous cytoplasmic roles. However, actin may interact with other actin-related proteins to regulate its dynamic properties to function in nucleo-cytoplasmic shuttling, chromatin remodeling, gene splicing expression regulation (Castano et. al., 2012; Rando et al. 2000; Vartiainen 2008) and maybe stress tolerance. In *Arabidopsis*, Abu-Abied et al. (2006) identified two cytoskeleton-interacting proteins, ERD10 and TCH2, for actin fiber association in rat fibroblasts by heterologous expression of yellow fluorescent protein fusion cDNA library from *Arabidopsis*. ERD10 (for early response to dehydration) belongs to a member of the dehydrin family, and TCH2 that is a touch-induced calmodulin-like protein could bind actin either directly or indirectly *in vitro*. Interestingly, in *Nicotiana benthamiana* cells, overexpression of ERD10 conferred resistance to latrunculin-mediated disruption of actin filaments. How ERD10 interacts with actin and different actin-binding proteins remains unknown. From previous proteomics research and gene expression analyses of the *OsADF* gene family, several OsADF proteins and genes were found induced under various abiotic stresses. These OsADFs may interact with actins or other proteins to play important regulatory roles in rice abiotic stress tolerance. Further screening of OsADF interacting proteins is needed to dissect the possible relationship between actin remodeling and the physiological function triggered by different stresses.

Meanwhile, recent growing evidence indicates that the rearrangement of a plant’s cytoskeleton can be a target for numerous stress signaling chains, such as touch, gravity, cold, salt, osmotic pressure and pathogen attacks (Abdrakhamanova et al. 2003; Engler et al. 2010; Nick, 2008; Wang et al. 2011). Wang et al. (2010) showed that assembly of salt stress-induced actin filament (AF) is a crucial factor involved in salt stress tolerance of Arabidopsis. The disruption of actin dynamics under salt stress was further demonstrated to coincide with increased reactive oxygen species levels in *Arabidopsis* root tip (Liu et al. 2012). In maize root, osmotic stress (PEG treatment) affected the fine structure of microtubule assembly, which was accompanied by increased ABA accumulation. Use of a microtubule destabilizer (e.g., oryzalin) or stabilizer (e.g., taxol) could stimulate ABA biosynthesis and increase osmotic stress tolerance (Lu et al. 2007). The participation of ADF in cytoskeleton rearrangement may represent as a new plant abiotic-stress tolerance regulation mechanism.

## Conclusions

In this study, we characterized the gene expression profile of the entire *OsADF* gene family with bioinformatics analysis of public microarray data and RT-PCR experiments. Then we focused on OsADF1 and 3 genes to determine their corresponding promoter activities and subcellular distribution. Finally by ectopically expressing OsADF3 gene in Arabidopsis, we investigated the responses of transgenic plants to various abiotic stresses. The results showed that *OsADF* genes expressed differentially in various rice tissues and under ABA or abiotic stress treatments (Figures 1 and 2). OsADF1 and OsADF3 proteins were located in the nucleus and expressed specifically in vascular tissues (Figures 3 and 4). After ABA and various abiotic stress treatments, OsADF3 GUS activity was further enhanced in lateral roots and root tips (Figure 5). *OsADF3*-heterologous transgenic *Arabidopsis* showed increased drought stress tolerance and up-regulation of many downstream drought-tolerant responsive genes (Figures 6, 7 and 8). Taken together, this study provides an example to demonstrate the role of *OsADF3* under drought and osmotic stresses and would benefit our further understanding of the function of rice OsADF gene family.

## Methods

### Plant materials, growth conditions and treatments

Seeds of rice *O*. *sativa* L. cv. Tainung 67 (TNG 67) were surface-sterilized in 2% sodium hypochloride followed by sterile water washes. Seeds were germinated at 37°C in the dark for 2 days and grown on Kimura B nutrient solution, pH 4.8 (Ma et al., 2001), in natural light at 30/25°C (daily/light). Twelve-day-old seedlings underwent treatment with different abiotic stresses, including 4°C for 6 hr; 10 μM ABA for 6 hr (light intensity ~250 μmol m^-2^ s^-1^, humidity ~60%); and 200 mM NaCl for 6 hr. Drought stress was imposed by inducing natural wilting with no nutrient solution for 2.5 hr (relative water loss at least < 50%). After treatments, fresh samples were harvested, immediately frozen in liquid N_2_ and stored at −80°C.

Arabidopsis seeds (ecotype Columbia-0) were surface sterilized in 2% sodium hypochloride with 0.05% Tween 20 followed by sterile water washes. Seeds were plated on 1/2 Murashige and Skoog (MS) mineral salts containing 1% sucrose with 0.3% phytagel for 4°C for 2 days. Seedlings were transferred to 16 h light/8 hr dark at 28°C with a light intensity of 80 nmole s^-1^ m^-2^. For the germination assays, approximately 30 to 35 seeds from wild type and transgenic plants were sown in triplicate on 1/2 MS medium with different concentrations of mannitol (0–300 mM). The seed germination rate was recorded as radicle emergence after seed sowing on 1/2 MS medium in the presence of mannitol (0–300 mM) for 5 days. For measurement of root growth, 1-week-old wild type and three T4 transgenic seedling lines (n = 6) were grown on 1/2 MS medium containing different concentrations of mannitol (0–300 mM) for 7 days. The primary root length was measured by use of Image J v1.39u (http://rsb.info.nih.gov/ij/). For drought stress tolerance analysis, water was withheld from 3-week-old wild type and transgenic lines grown in soil for 12 days. Photographs were taken 0, 5 and 8 days after watering was resumed.

### RT-PCR and Real-Time PCR analysis

Total RNA was extracted by the Trizol reagent method (Invitrogen, USA). To remove genomic DNA, total RNA was treated with Turbo DNase I (Ambion, TX, USA) for 30 min at 37°C. To ensure complete elimination of contaminated DNA, samples underwent PCR, with DNase I-treated RNA used as a template. For each sample, 2 μg total RNA was reverse transcribed into first-strand cDNA with use of an oligo dT primer (Superscript III 1st Strand Synthesis Kit, Invitrogen). An aliquot of the first-strand cDNA mixture corresponding to 100 ng total RNA was used as a template. PCR amplification was 94°C for 3 min, 35 cycles of 94°C for 30 sec, 55°C for 30 sec, 72°C for 30 sec, then 72°C for 3 min. To increase the specificity of gene amplification, primer sets were designed with use of Vector NTI (v9.0) with the 3’UTR sequence for each OsADF gene, except for *OsADF7*, *OsADDF8a* and *OsADF8b*, whose primers matched the coding region. The PCR amplicons were sequenced and used for a BLAST search of the GenBank database to ensure no significant homology. The specific primers are in Additional file 7: Table S1. For semi-quantitative RT-PCR, the rice ubiquitin gene (*OsUBI*, D12629) was used as an internal control for standardizing the amount of input cDNA template and as a reference to normalize the relative expression of target mRNA. The amplified PCR products were resolved on a 3% agarose gel and stained with ethidium bromide. The intensity of the bands in the gel was visualized by use of the SynGene gel documentation system and analyzed with use of Genetools (Syngene, MD, USA).

For real-time PCR analysis, an aliquot of the first-strand cDNA mixture corresponding to 10 ng total RNA was used as a template. Real-time PCR involved the SYBR Green PCR master mix (Applied Biosystems, USA) with the ABI7500 real-time PCR system. Gene-specific primer sequences are in Additional file 7: Table S1. Relative mRNA expression of target genes was normalized to that of an internal control, *AtUBC10* (At5G53300), and calculated as 2 ^-ΔΔCt^ in comparison to unstressed seedlings (Livak and Schmittgen, 2001). All analyses involved 3 replicates of amplifications with 3 independent batches of total RNA samples. Results are shown as means ± standard errors from at least 3 independent experiments.

### Construction of p*ubi*::*OsADF1*-*GFP*, p*ubi*::*OsADF3*-*GFP*, *p*_*OsADF1i*_::*GUS* and *p*_*OsADF3i*_::*GUS* expression plasmids

The p*ubi*::*GFP*, p*35S*::*HPT* and pCYH10 vectors (from Dr. Chwan-Yang Hong, National Taiwan University) were used for chimeric gene construction, transient protein subcellular localization and production of transgenic rice plants. The pCYH10 vector is a promoter-less vector that contains a complete coding sequence of GUS. Full-length cDNAs of *OsADF1* and *OsADF3* were amplified from TNG 67 rice by RT-PCR with the primers ADF1-F (5’-ggatcc ATGTCGAATTCGGCGTCGGGAAT-3’), ADF1-R (5’-ctcgag GAGGGCTCGCGACTTGACGATGT-3’); and ADF3-F (5’-ggatcc ATGGCGAACGCGACGTCGGGTGT-3’) and ADF3-R (5’-ctcgag GGAGGTGTGGTCCTTGAGCACGT-3’). The open reading frame (ORF) of *GPF* was amplified with GFP-F (5’-ctcgag GTGAGCAAGGGCGAG-3’) and GFP-R (5’-actagt CTACTTGTACAGCTCGTCCA-3’). The restriction sites of *BamHI* GGATCC, *XhoI* CTCGAG and *SpeI* ACTAGT (sequence in small letters underlined) were added to the end of the primer for conventional cloning. The vector of p*ubi*::*GFP* was digested with *BamHI* and *SpeI* to remove the GFP cDNA fragment. The amplified fragments of OsADF1, OsADF3 and GFP genes were digested with *BamHI*, *XhoI* or *SpeI* and cloned in-frame into the *BamHI* and *SpeI* restriction sites of the p*ubi*::*GFP* vector to generate p*ubi*::*OsADF1*-*GFP* and p*ubi*::*OsADF3*-*GFP*. For vector construction for GUS activity assay, the 1.7- and 1.6-kb promoter DNA fragments including the upstream ATG start codon and the first intron of *OsADF1* and *OsADF3*, respectively, were amplified by PCR with the primer pairs pADF1-F (5’-ggtacc GTCAGGGAAGCATGCCAAGTGC-3’) with a *KpnI* site, pADF1-R with a *BamHI* site (5’-ggatcc CTTACATATCCCCACAACATAC-3’); and pADF3–F (5’-cccggg CTGGGGATAAACGGGGCCTCTA-3’) with an *SmaI* site and pADF3-R (5’-cccggg CTGCACAAACACACGCATAAAG-3’) with an *SmaI* site. The amplified genomic DNAs for *pOsADF1* and *pOsADF3* were digested separately by *KpnI*/*BamHI* and *SmaI* and ligated into the corresponding cut sites of pCYH10. The whole construct was introduced into pCAMBIA 1302 to generate *p*_*OsADF1i*_::*GUS* and *p*_*OsADF3i*_::*GUS* with the first intron, respectively, and used for transformation into rice.

### Particle bombardment assay and histochemical staining of GUS activity

Transient expression assay was performed by particle bombardment with onion epidermal cells and the PDS-1000/He biolistic particle delivery system (Bio-Rad, CA, USA) as described (Varagona et al., 1992). At 24 hr after the p*ubi*::*OsADF1GFP* or p*ubi*::*OsADF3GFP* construct was delivered into onion epidermal cells, GFP fluorescence was visualized under a Zeiss Axioplan fluorescence microscope. Tissues at different developmental stages from T_0_ and seedlings from T_1_ transgenic rice carrying the introduced genes were collected for GUS activity analysis. GUS staining was detected after incubating different samples at 37°C in a solution with 1 mM X-gluc and 0.5 mM potassium ferricyanide for 16 to 18 hr. After staining, chlorophyll in the tissue was removed with 95% ethanol.

### Generation of transgenic rice and *Arabidopsis* plants

Rice transformation involved introducing *p*_*OsADF1i*_::*GUS* or *p*_*OsADF3i*_::*GUS* by electroporation with *Agrobacterium tumefaciens* strain EHA101. The callus from an immature rice embryo (cv. TNG 67) was transformed by *Agrobacterium*-mediated transformation as described (Hiei and Komari, 2008). Transformed calli were then selected on N6 medium that contained 50 μg l^-1^ hygromycin. For overexpression of *OsADF3* in *Arabidopsis*, the full-length cDNA of *OsADF3* was amplified with the primers ADF3-F (5’-ggatcc ATGGCGAACGCGACGTCGGGTGT-3’) and ADF3-R2 (5’-ggatcc GGAGGTGTGGTCCTTGAGCACGT-3’), then the amplified ORF fragment was subcloned into a BamHI-digested p*35S*::*HPT* vector with the HPT cDNA fragment removed. The resulting plasmid was introduced into pCAMBIA 1302 binary vector to obtain the p*35S*::*OsADF3* construct used for *Agrobacterium* (strain GV3101)-mediated gene transformation in *Arabidopsis thaliana* ecotype Col-0 transformation by the floral dip method (Clough and Bent 1998). Finally, to obtain T_4_ homozygous lines of *35S*::*OsADF3* overexpression transgenic plants for drought/osmotic stress response analysis, 6 independent transgenic lines were selected by planting seeds on 1/2 MS media containing 25 mg/L hygromycin B (InvivoGen, USA). Three homozygous lines of transgenic plants were chosen for further study.

## Electronic supplementary material

Additional file 1:**Table S2.** Members of the OsADF gene family and their predicted gene structures. (PDF 49 KB)

Additional file 2:**Figure S1.** Amino acid sequence alignment of actin depolymerizing factor (ADF) proteins from Arabidopsis and rice. The deduced amino acid sequences of different ADF parologues from Arabidopsis, rice and other species were aligned by use of Align X. Grey or dark shading with letters represent similar or identical amino acid residues. To allow for maximal sequence alignment, dashes were inserted in the sequence. The putative phosphorylated serine amino residue is marked by * and the site (KRXHP) for a putative nuclear localization signal (NLS) transport is boxed. The highest identity was found between the isoforms *OsADF8* and *OsADF10* (94%), *OsADF1* and *OsADF6* (90%), *OsADF1* and *OsADF9* (78%), *OsADF6* and *OsADF9* (77%), *OsADF2* and *OsADF11* (77%). The sequences were derived from the following accession numbers (Genebank ID) : *AtADF1*, At3g46010, *AtADF2*, At3g46000, *AtADF3*, At5g59880, *AtADF4*, At5g59890, *AtADF5*, At2g16700, *AtADF6*, At2g31200, *AtADF7*, At4g25590, *AtADF8*, At4g00680, *AtADF9*, At4g34970, *AtADF10*, At5g052360, *AtADF11*, At1g01750, *OsADF1*, LOC_Os02g44470, *OsADF2* LOC_Os03g56790, *OsADF3*, LOC_Os03g60580, *OsADF4*, LOC_Os03g60590, *OsADF5*, LOC_Os03g13950, *OsADF6*, LOC_Os04g46910, *OsADF7*, LOC_Os05g02250, *OsADF8a*, AP004760, *OsADF8b*, AP006344, *OsADF9*, LOC_Os07g30090, *OsADF10*, LOC_Os10g37670, *OsADF11*, LOC_Os12g43340. (PDF 449 KB)

Additional file 3:**Figure S2.** Phylogenetic analysis of ADFs from *Arabidopsis* and rice. The unrooted tree was constructed with the deduced amino acid sequences from *Oryza sativa* (*Os*) and *Arabidopsis thaliana* (*At*) using the CLUSTALW 1.83 software and displayed with the Treeview program. The minimal bootstrap cut value was set at 700. The length of tree represents the extent of diversity and the scale bar corresponds to a distance of 0.1 amino acid substitutions per alignment position. The *Arabidopsis*-rice phylogenetic tree showed 6 groups: (*OsADF11*, *OsADF2* and *AtADF6*), B (*OsADF5*, *AtADF5* and *AtADF9*), C (*OsADF9*; *AtADF7*, *8*, *10*, *11*; *OsADF1* and *OsADF6*), D (*AtADF1*, *2*, *3* and *4*), E (*OsADF3* and *OsADF4*), and F (*OsADF10*, *8* and *7*). *OsADF7* was distinguished from the others by its extraordinary genetic distance. (PDF 35 KB)

Additional file 4:**Figure S3.** Analysis of the putative ABA-responsive element (ABRE), dehydration-responsive element/C-repeat (DRE/CRT), and low-temperature response element (LTRE) cis-acting elements present in the 1-kb promoter regions of rice OsADFs by use of the PLACE dataset. The locations of various elements are labeled. RT-PCR analysis of stress- or ABA-induced OsADF gene expression in 12-day-old rice seedlings is marked with checks to the right of the corresponding genes (C: cold, A: ABA, S: salt, D: drought). All OsADF genes except OsADF7 contained at least 1 of the 3 types of cis-acting elements (ABRE: OsADF1, 3 and 9; LTRE: OsADF10; ABRE and LTRE: OsADF2, 5, 6 and 11, ABRE, LTRE and DRE: OsADF4, 8a and 8b). (PDF 28 KB)

Additional file 5:**Figure S4.** RT-PCR determination of rice actin depolymerizing factors (OsADFs) experssion in different tissues of rice (Tainung 67) at various developmental stages from 12-, 45- to 90-day-old. (A) Transcripts of OsADFs in shoot and root of 12-day-old rice seedlings and in leaf blade, leaf sheath and root at early tillering stage (45 days old). (B) Transcript levels of OsADFs in root, stem, leaf blade, leaf sheath, spikelet at heading stage (90 days old). OsADF expression is relative to that of the rice ubiquitin gene OsUBI (D12629) used as an internal control. (PDF 99 KB)

Additional file 6:**Figure S5.** RT-PCR determination of *OsADFs* expression under various abiotic stresses and abscissic acid (ABA) treatment in root or shoot of 12-day-old rice seedlings. The numbers on the right refer to the PCR cycles. St: shoot; R: root; K: control; C: cold; S: salt; D: drought; A: ABA. OsADF expression is relative to that of OsUBI (D12629) used as an internal control. (PDF 99 KB)

Additional file 7:**Table S1.** Gene-specific primer pairs used for RT-PCR (right) or real-time PCR (left) analysis of mRNA expression of rice actin depolymerizing factor (*OsADF*) in rice and in *Arabidopsis*. (PDF 39 KB)

Below are the links to the authors’ original submitted files for images.

Authors’ original file for figure 1

Authors’ original file for figure 2

Authors’ original file for figure 3

Authors’ original file for figure 4

Authors’ original file for figure 5

Authors’ original file for figure 6

Authors’ original file for figure 7

Authors’ original file for figure 8

## Abbreviations

ABA: Abscisic acid
ABRE: ABA-responsive element
ADF: Actin depolymerizing factor
DRE/CRT: Dehydration-responsive element/C-repeat
GEO: Gene expression omnibus
GFP: Green fluorescent protein
GUS: β-glucuronidase
LTRE: Low-temperature responsive element
ORF: Open reading frame.
