# Supplementary material for: Comprehensive analysis of differentially expressed rice actin depolymerizing factor gene family and heterologous overexpression of OsADF3 confers Arabidopsis Thaliana drought tolerance
Source: Rice (N Y). 2012 Nov 27;5:33. doi: 10.1186/1939-8433-5-33 (PMC4883719; doi:10.1186/1939-8433-5-33)
Supplement: Supplementary file 1 — Additional file 1:Table S2. Members of the OsADF gene family and their predicted gene structures. (PDF 49 KB) [file 12284_2012_34_MOESM1_ESM.pdf]

Additional file 3

Supplementary Table S2. Members of the OsADF gene family and their predicted gene structures.

Gene name	Chr.	Genebank ID	TIGR Locus No.	KOME clone	Predicted [a] protein length	Gene structure [b]
OsADF1	2	AP004037	LOC_Os02g44470	AK069605	139 (158)	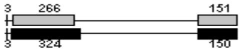
OsADF2	3	AC084320	LOC_Os03g56790	AK073162	145	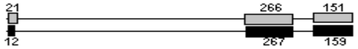
OsADF3	3	AC104433	LOC_Os03g60580	AK241081	150	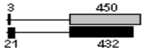
OsADF4	3	AC104433	LOC_Os03g60590	AK058941	139	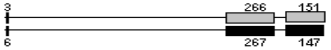
OsADF5	3	AC134239	LOC_Os03g13950	AK104056	143 (94)	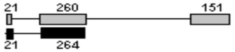
OsADF6	4	AL606647	LOC_Os04g46910	AK069329	139 (419)	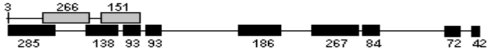
OsADF7	5	AC093921	LOC_Os05g02250	AK102177	141 (132)	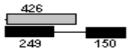
OsADF8a	6	AP004760	ND	ND	146 (416)	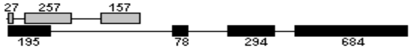
OsADF8b	7	AP006344	ND	ND	146 (416)	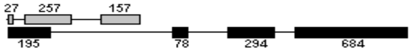
OsADF9	7	AP005465	LOC_Os07g30090	AK072662	139 (145)	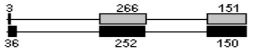
OsADF10	10	AC051634	LOC_Os10g37670	AK121749	151 (153)	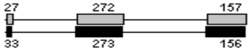
OsADF11	12	AC027133	LOC_Os12g43340	AK121150	145	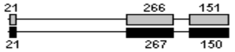

[a.] Bracketed number indicates deduced amino acid length of *OsADF* previously published by Feng et al. (2006). Number without brackets indicates the length of *OsADF* amino acids predicted by this study.

[b.] Gene structure of the *OsADFs* identified by Feng et al. (2006) (in black) and by this study (in grey). Rectangles and lines represent exons and introns, respectively. The number above or below the box represents the length in bp of each exon. Except for *OsADF6*, *7*, *8a* and *8b*, all gene structures were compared and shown in corresponding position to highlight variation in size. ND: not identified.

(Supplementary Table S2, Huang *et al.*, 2012)
