# Supplementary figures and images for: Comprehensive analysis of differentially expressed rice actin depolymerizing factor gene family and heterologous overexpression of OsADF3 confers Arabidopsis Thaliana drought tolerance
Source: Rice (N Y). 2012 Nov 27;5:33. doi: 10.1186/1939-8433-5-33 (PMC4883719; doi:10.1186/1939-8433-5-33)

Additional file 4

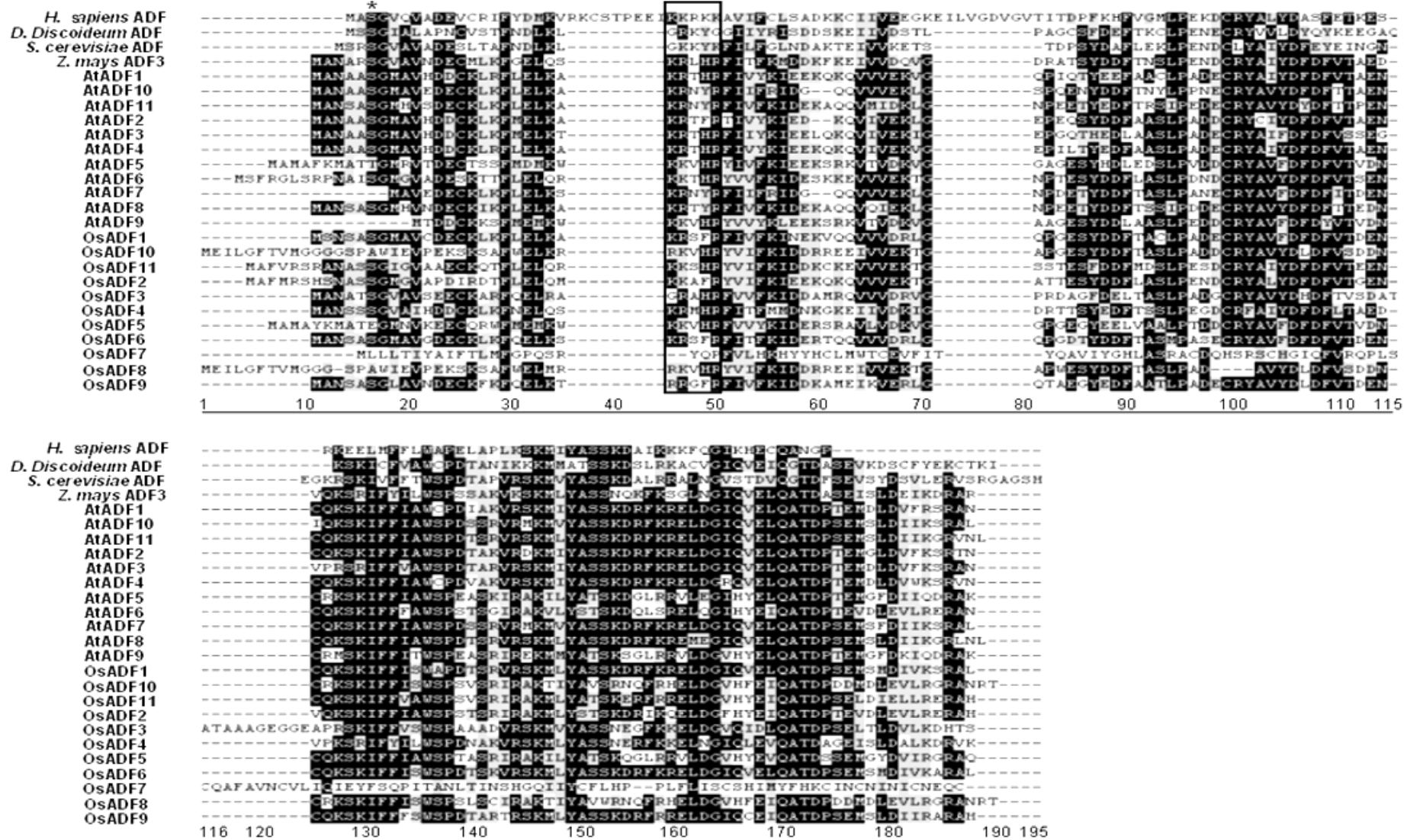

(Supplementary Fig. S1., Huang *et al.*, 2012)

Supplement: Supplementary file 2 — Additional file 2:Figure S1. Amino acid sequence alignment of actin depolymerizing factor (ADF) proteins from Arabidopsis and rice. The deduced amino acid sequences of different ADF parologues from Arabidopsis, rice and other species were aligned by use of Align X. Grey or dark shading with letters represent similar or identical amino acid residues. To allow for maximal sequence alignment, dashes were inserted in the sequence. The putative phosphorylated serine amino residue is marked by * and the site (KRXHP) for a putative nuclear localization signal (NLS) transport is boxed. The highest identity was found between the isoforms OsADF8 and OsADF10 (94%), OsADF1 and OsADF6 (90%), OsADF1 and OsADF9 (78%), OsADF6 and OsADF9 (77%), OsADF2 and OsADF11 (77%). The sequences were derived from the following accession numbers (Genebank ID) : AtADF1, At3g46010, AtADF2, At3g46000, AtADF3, At5g59880, AtADF4, At5g59890, AtADF5, At2g16700, AtADF6, At2g31200, AtADF7, At4g25590, AtADF8, At4g00680, AtADF9, At4g34970, AtADF10, At5g052360, AtADF11, At1g01750, OsADF1, LOC_Os02g44470, OsADF2 LOC_Os03g56790, OsADF3, LOC_Os03g60580, OsADF4, LOC_Os03g60590, OsADF5, LOC_Os03g13950, OsADF6, LOC_Os04g46910, OsADF7, LOC_Os05g02250, OsADF8a, AP004760, OsADF8b, AP006344, OsADF9, LOC_Os07g30090, OsADF10, LOC_Os10g37670, OsADF11, LOC_Os12g43340. (PDF 449 KB) [file 12284_2012_34_MOESM2_ESM.pdf]

Additional file 5

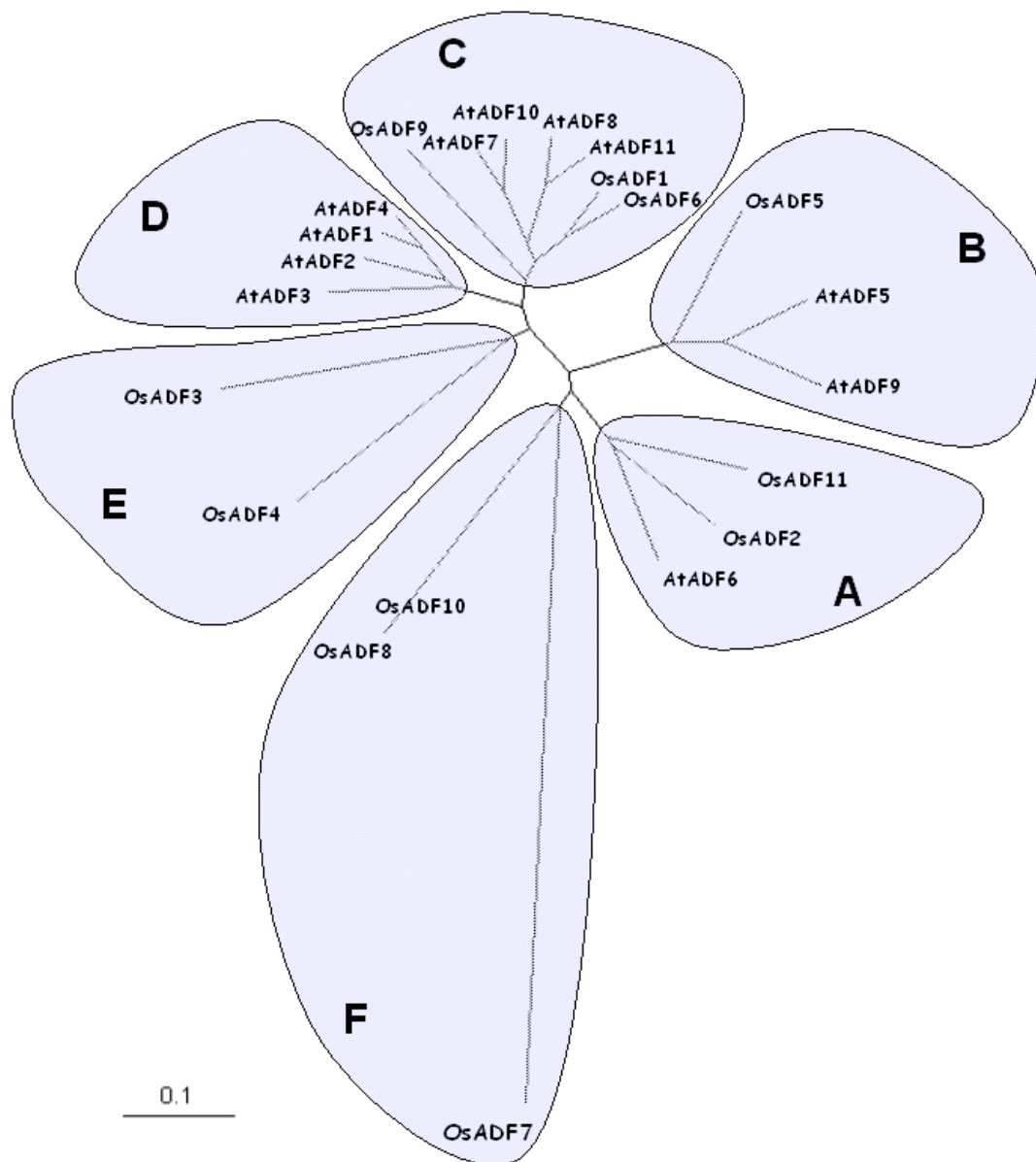

(Supplementary Fig.S2., Huang *et al.*, 2012)

Supplement: Supplementary file 3 — Additional file 3:Figure S2. Phylogenetic analysis of ADFs from Arabidopsis and rice. The unrooted tree was constructed with the deduced amino acid sequences from Oryza sativa (Os) and Arabidopsis thaliana (At) using the CLUSTALW 1.83 software and displayed with the Treeview program. The minimal bootstrap cut value was set at 700. The length of tree represents the extent of diversity and the scale bar corresponds to a distance of 0.1 amino acid substitutions per alignment position. The Arabidopsis-rice phylogenetic tree showed 6 groups: (OsADF11, OsADF2 and AtADF6), B (OsADF5, AtADF5 and AtADF9), C (OsADF9; AtADF7, 8, 10, 11; OsADF1 and OsADF6), D (AtADF1, 2, 3 and 4), E (OsADF3 and OsADF4), and F (OsADF10, 8 and 7). OsADF7 was distinguished from the others by its extraordinary genetic distance. (PDF 35 KB) [file 12284_2012_34_MOESM3_ESM.pdf]

Additional file 6

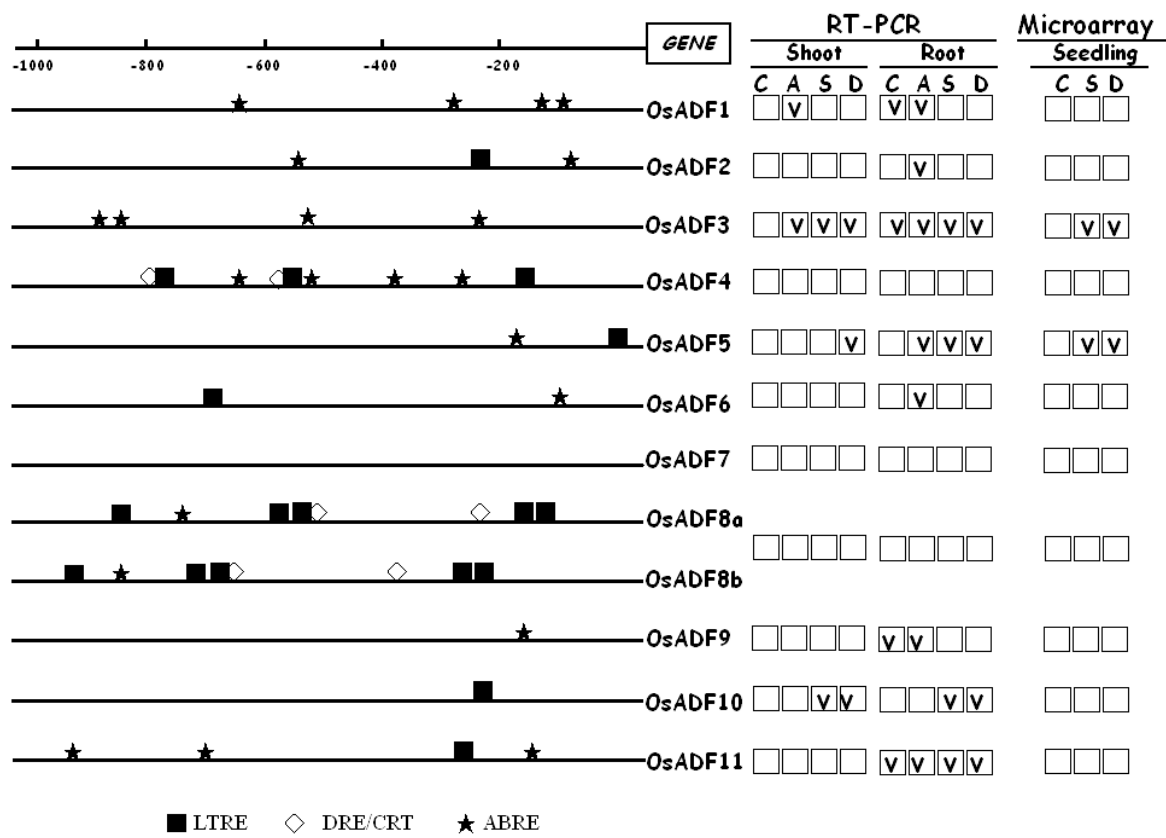

(Supplementary Fig. S3., Huang *et al.*, 2012)

Supplement: Supplementary file 4 — Additional file 4:Figure S3. Analysis of the putative ABA-responsive element (ABRE), dehydration-responsive element/C-repeat (DRE/CRT), and low-temperature response element (LTRE) cis-acting elements present in the 1-kb promoter regions of rice OsADFs by use of the PLACE dataset. The locations of various elements are labeled. RT-PCR analysis of stress- or ABA-induced OsADF gene expression in 12-day-old rice seedlings is marked with checks to the right of the corresponding genes (C: cold, A: ABA, S: salt, D: drought). All OsADF genes except OsADF7 contained at least 1 of the 3 types of cis-acting elements (ABRE: OsADF1, 3 and 9; LTRE: OsADF10; ABRE and LTRE: OsADF2, 5, 6 and 11, ABRE, LTRE and DRE: OsADF4, 8a and 8b). (PDF 28 KB) [file 12284_2012_34_MOESM4_ESM.pdf]

Additional file 7

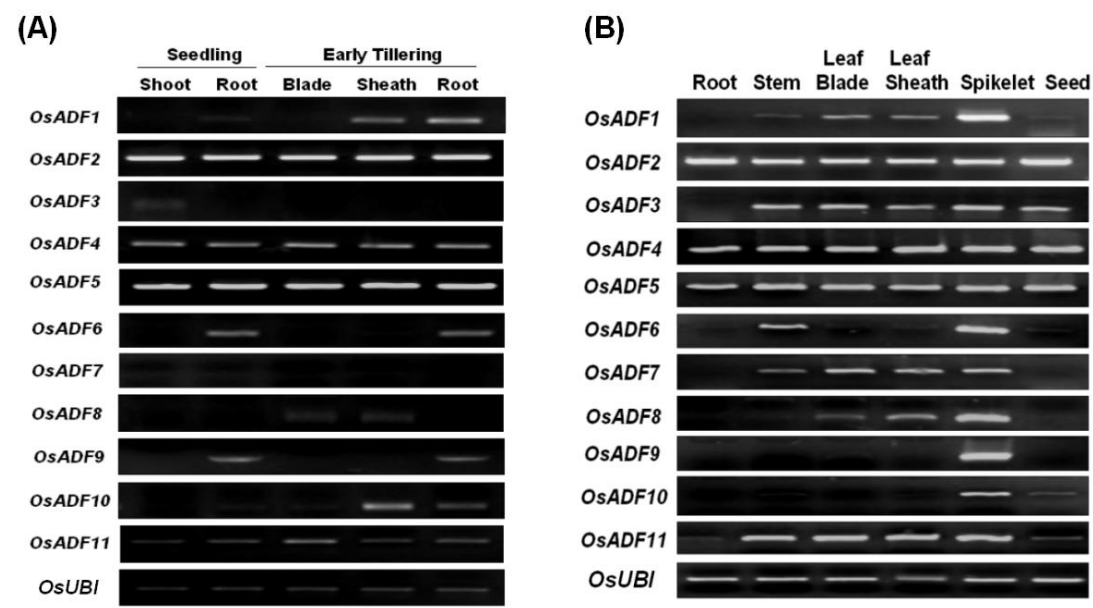

(Supplementary Fig. S4., *Huang et al.*, 2012)

Supplement: Supplementary file 5 — Additional file 5:Figure S4. RT-PCR determination of rice actin depolymerizing factors (OsADFs) experssion in different tissues of rice (Tainung 67) at various developmental stages from 12-, 45- to 90-day-old. (A) Transcripts of OsADFs in shoot and root of 12-day-old rice seedlings and in leaf blade, leaf sheath and root at early tillering stage (45 days old). (B) Transcript levels of OsADFs in root, stem, leaf blade, leaf sheath, spikelet at heading stage (90 days old). OsADF expression is relative to that of the rice ubiquitin gene OsUBI (D12629) used as an internal control. (PDF 99 KB) [file 12284_2012_34_MOESM5_ESM.pdf]

Additional file 8

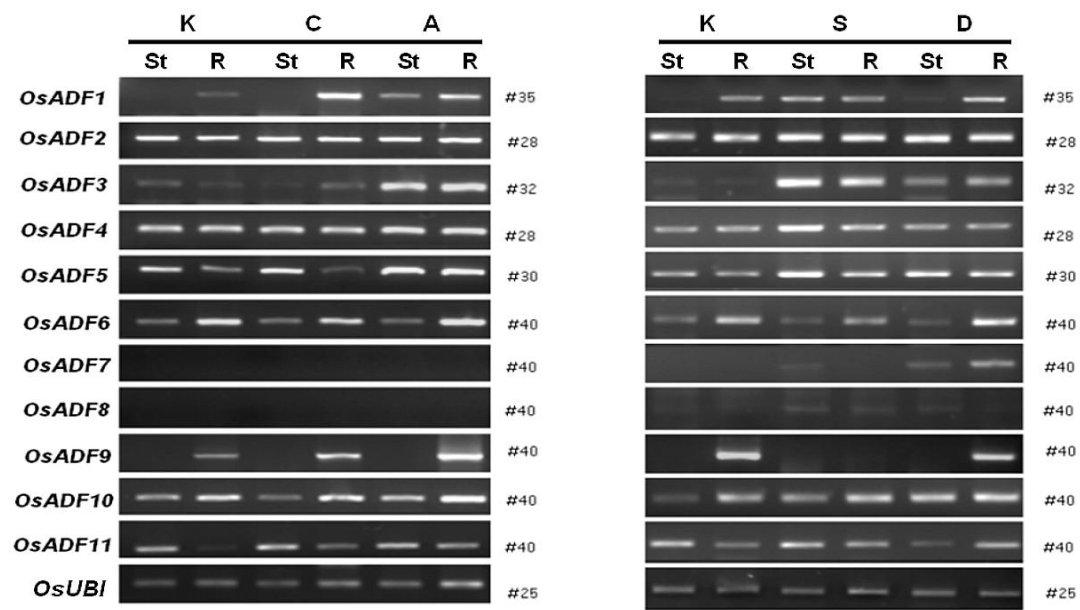

(Supplementary Fig. S5., *Huang et al.*, 2012)

Supplement: Supplementary file 6 — Additional file 6:Figure S5. RT-PCR determination of OsADFs expression under various abiotic stresses and abscissic acid (ABA) treatment in root or shoot of 12-day-old rice seedlings. The numbers on the right refer to the PCR cycles. St: shoot; R: root; K: control; C: cold; S: salt; D: drought; A: ABA. OsADF expression is relative to that of OsUBI (D12629) used as an internal control. (PDF 99 KB) [file 12284_2012_34_MOESM6_ESM.pdf]

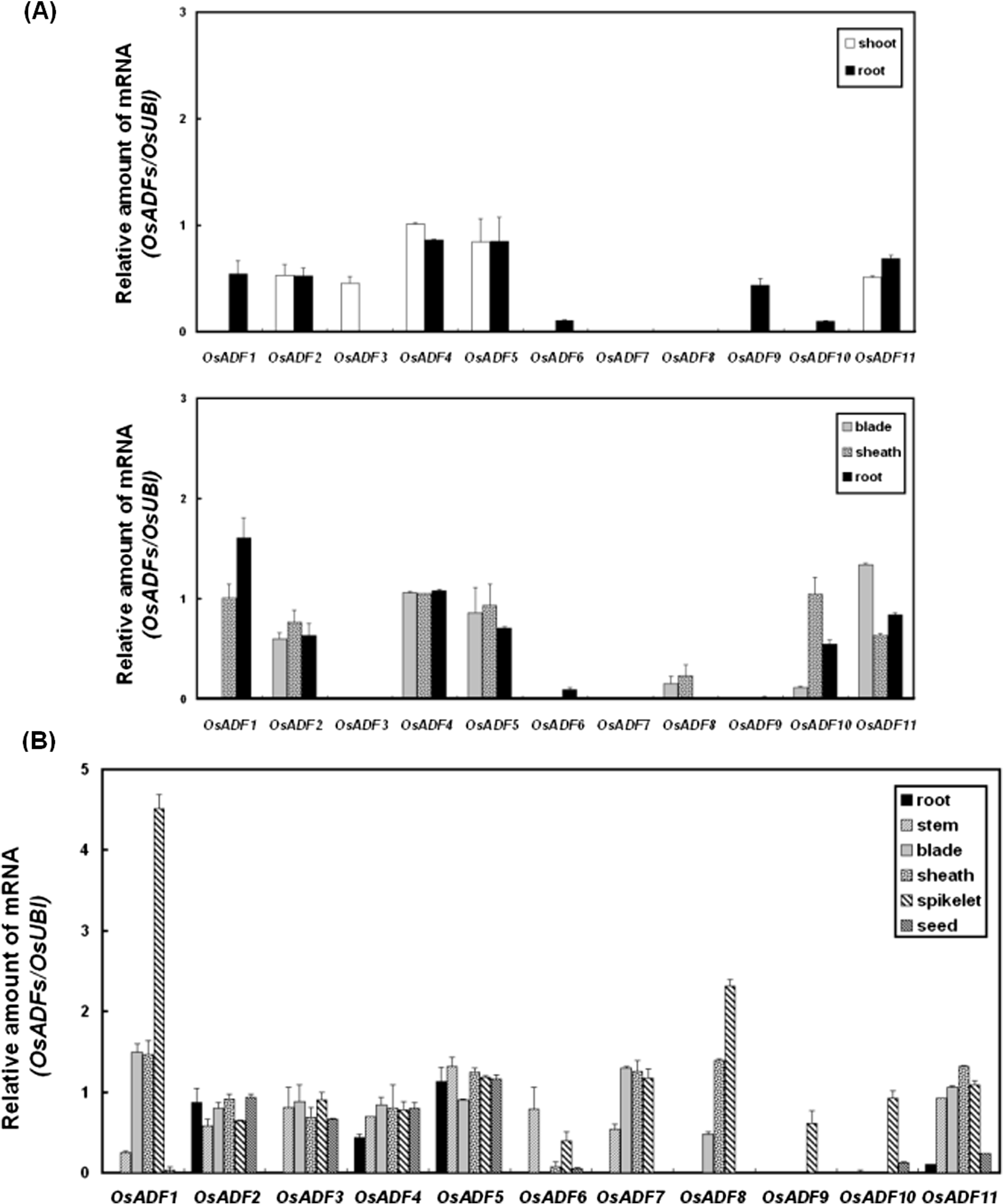

Supplement: Supplementary file 8 — Authors’ original file for figure 1 [file 12284_2012_34_MOESM8_ESM.tiff]

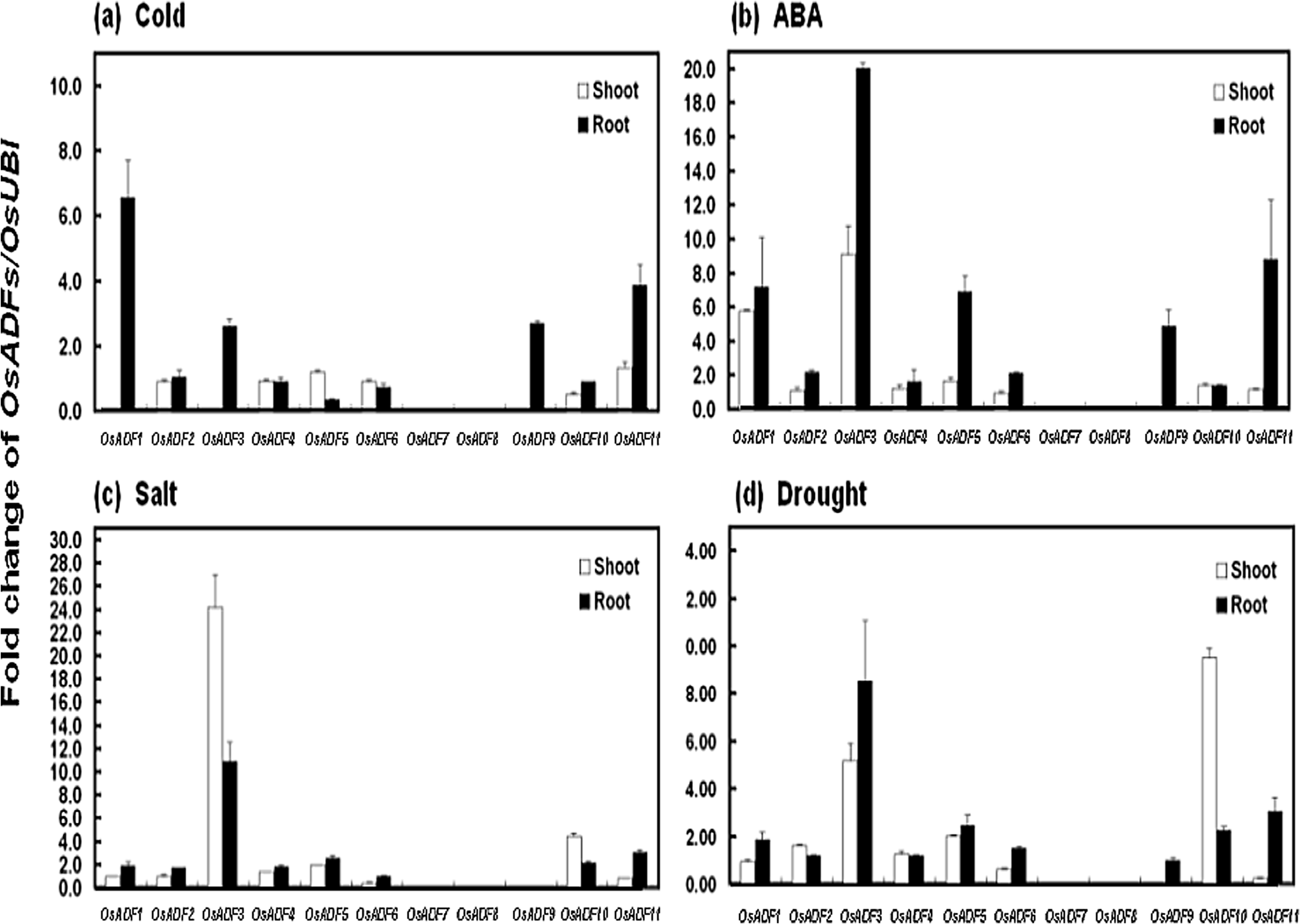

Supplement: Supplementary file 9 — Authors’ original file for figure 2 [file 12284_2012_34_MOESM9_ESM.tiff]

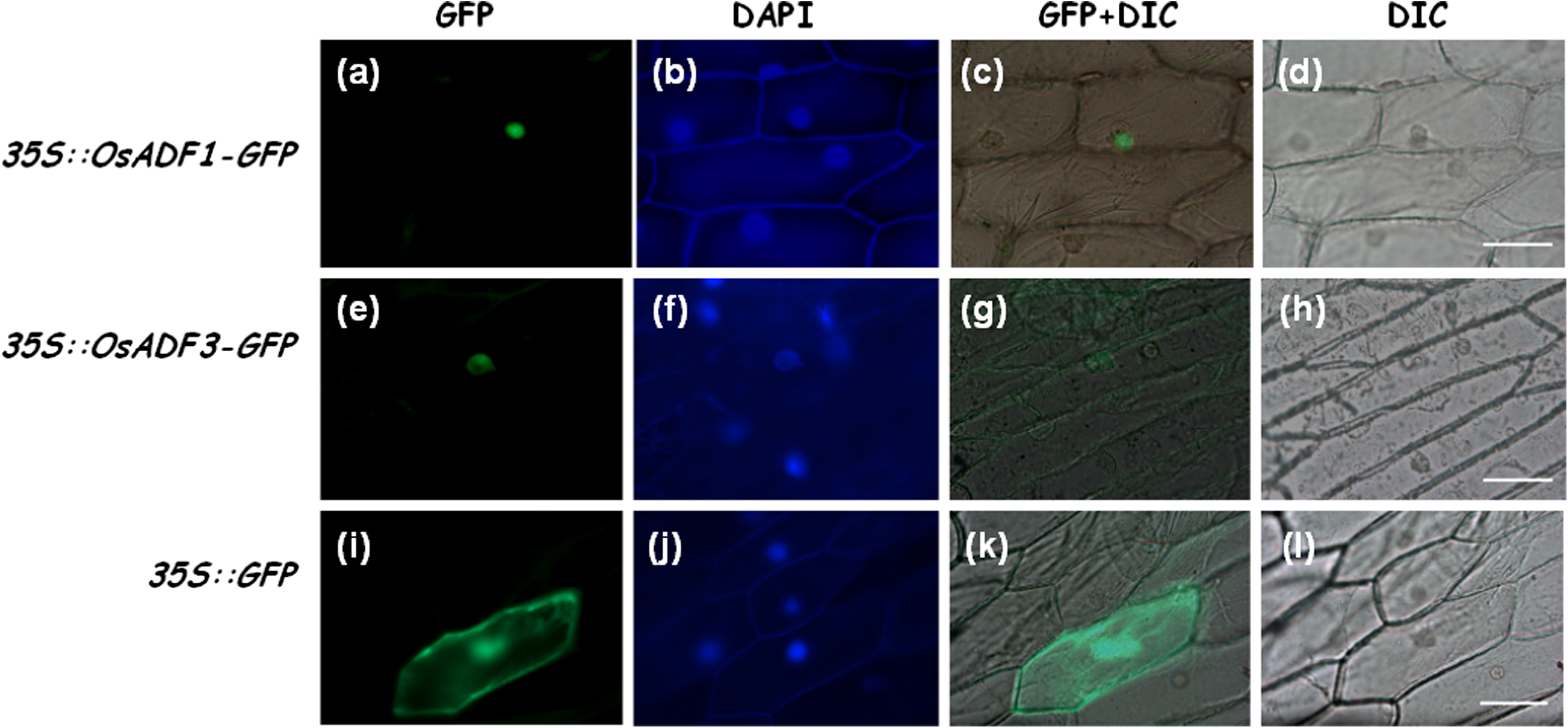

Supplement: Supplementary file 10 — Authors’ original file for figure 3 [file 12284_2012_34_MOESM10_ESM.tiff]

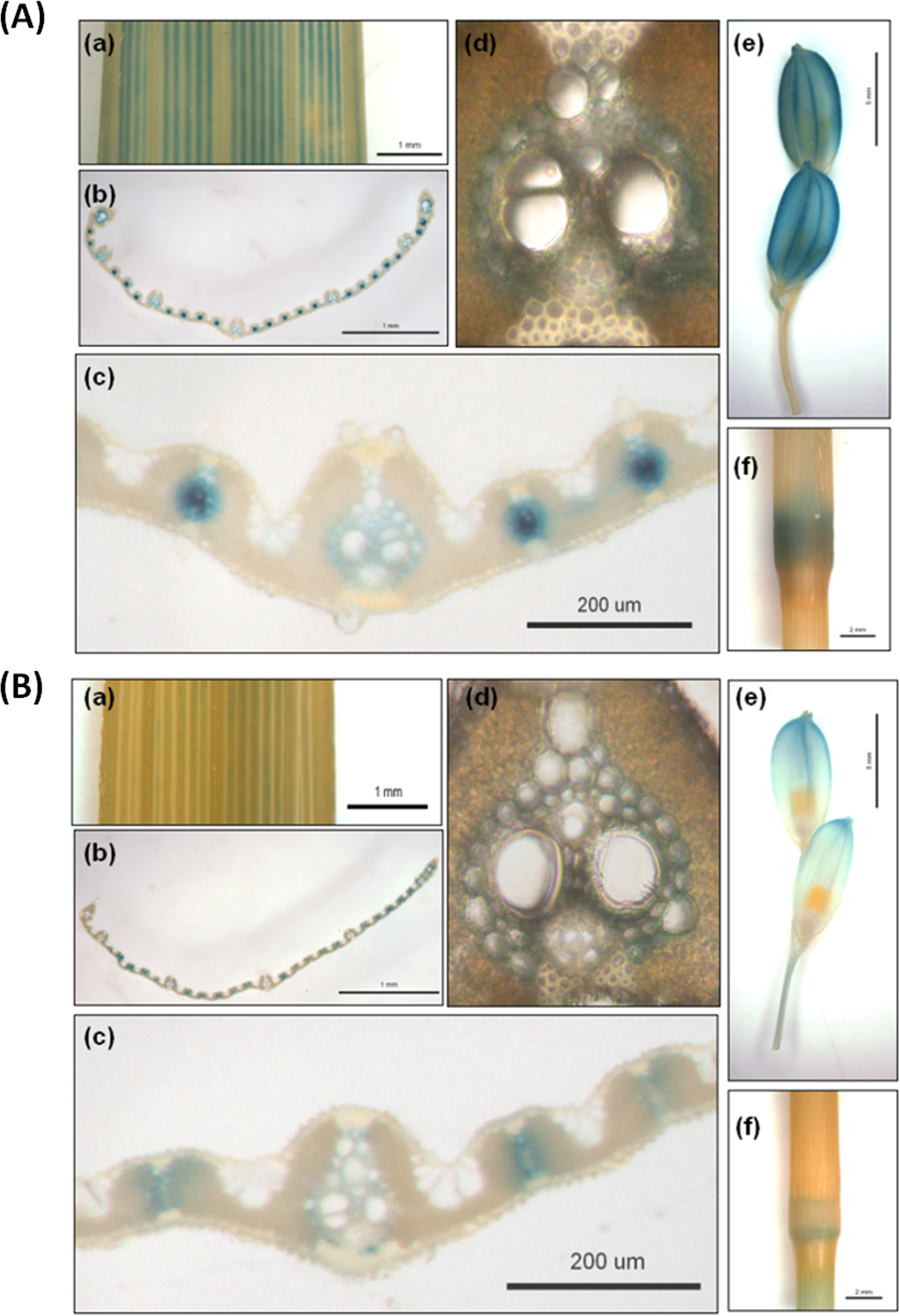

Supplement: Supplementary file 11 — Authors’ original file for figure 4 [file 12284_2012_34_MOESM11_ESM.tiff]

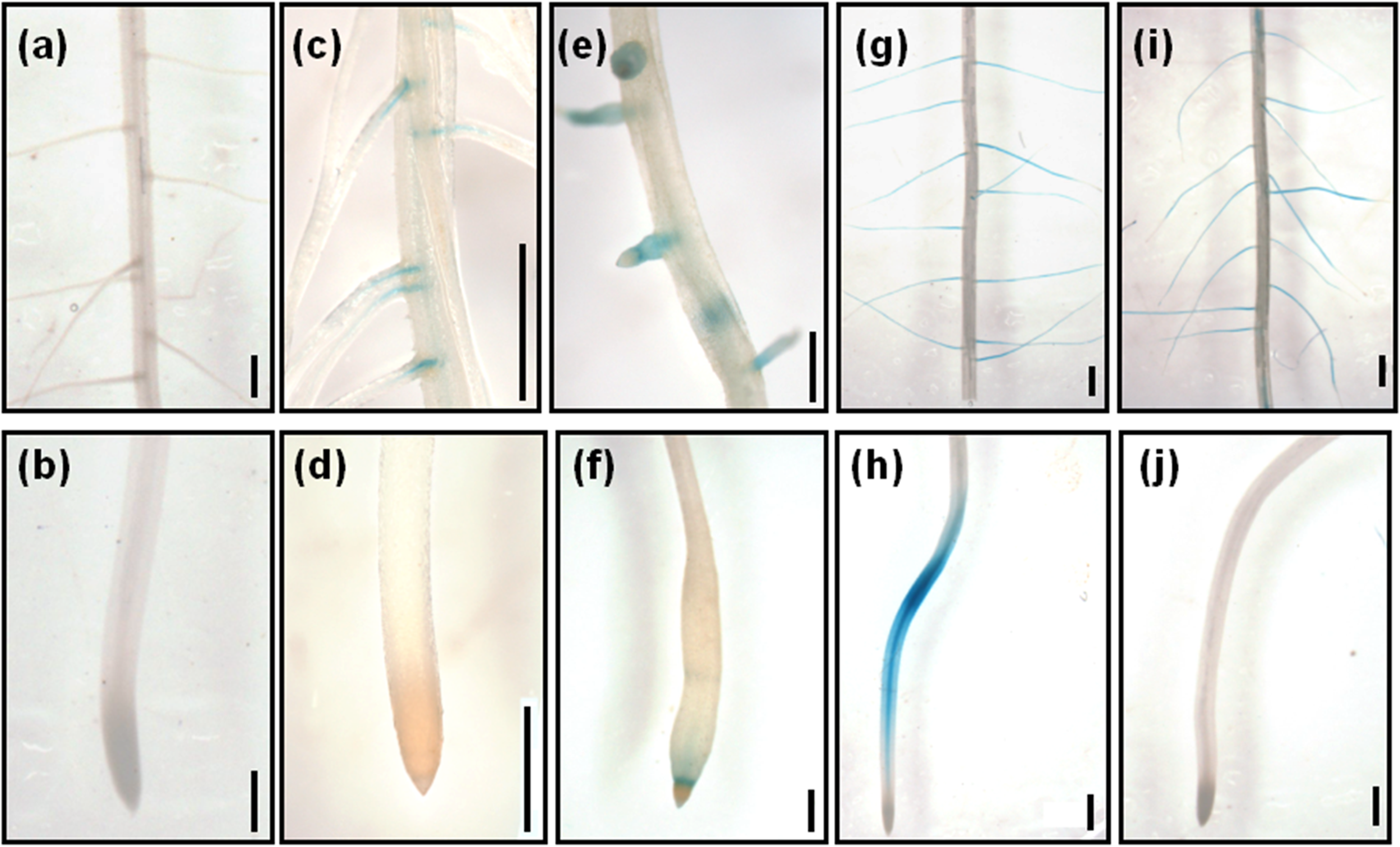

Supplement: Supplementary file 12 — Authors’ original file for figure 5 [file 12284_2012_34_MOESM12_ESM.tiff]

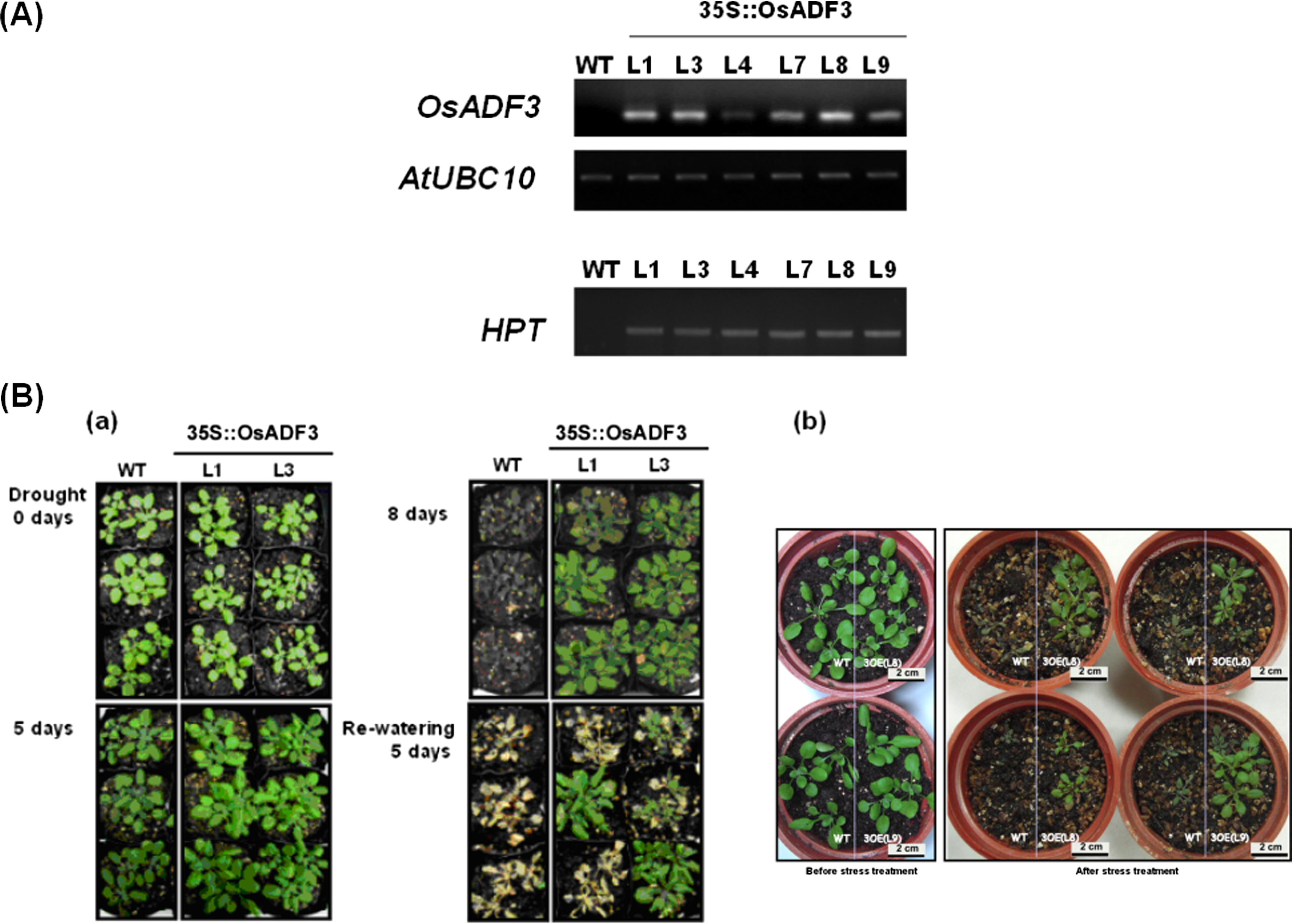

Supplement: Supplementary file 13 — Authors’ original file for figure 6 [file 12284_2012_34_MOESM13_ESM.tiff]

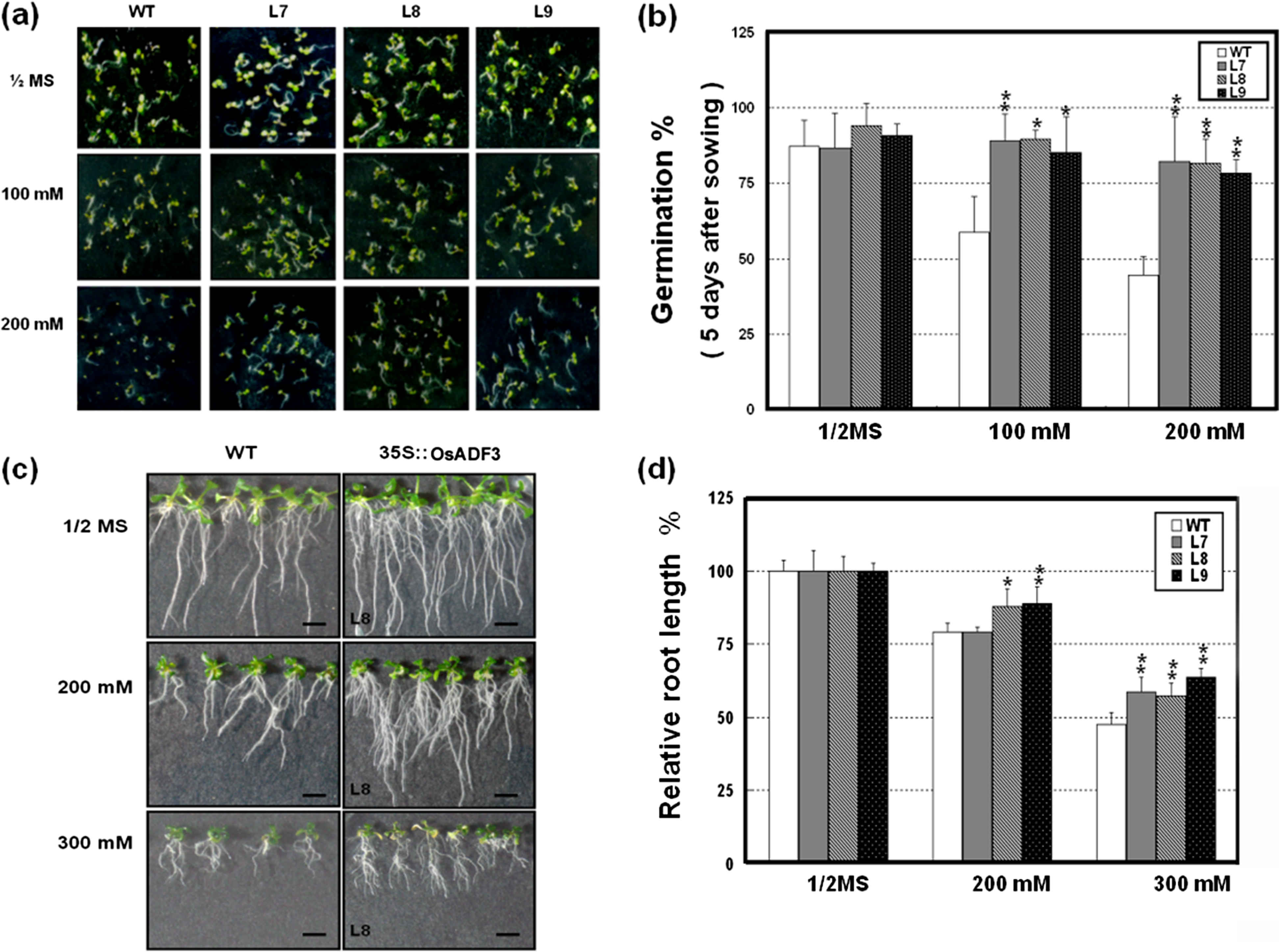

Supplement: Supplementary file 14 — Authors’ original file for figure 7 [file 12284_2012_34_MOESM14_ESM.tiff]

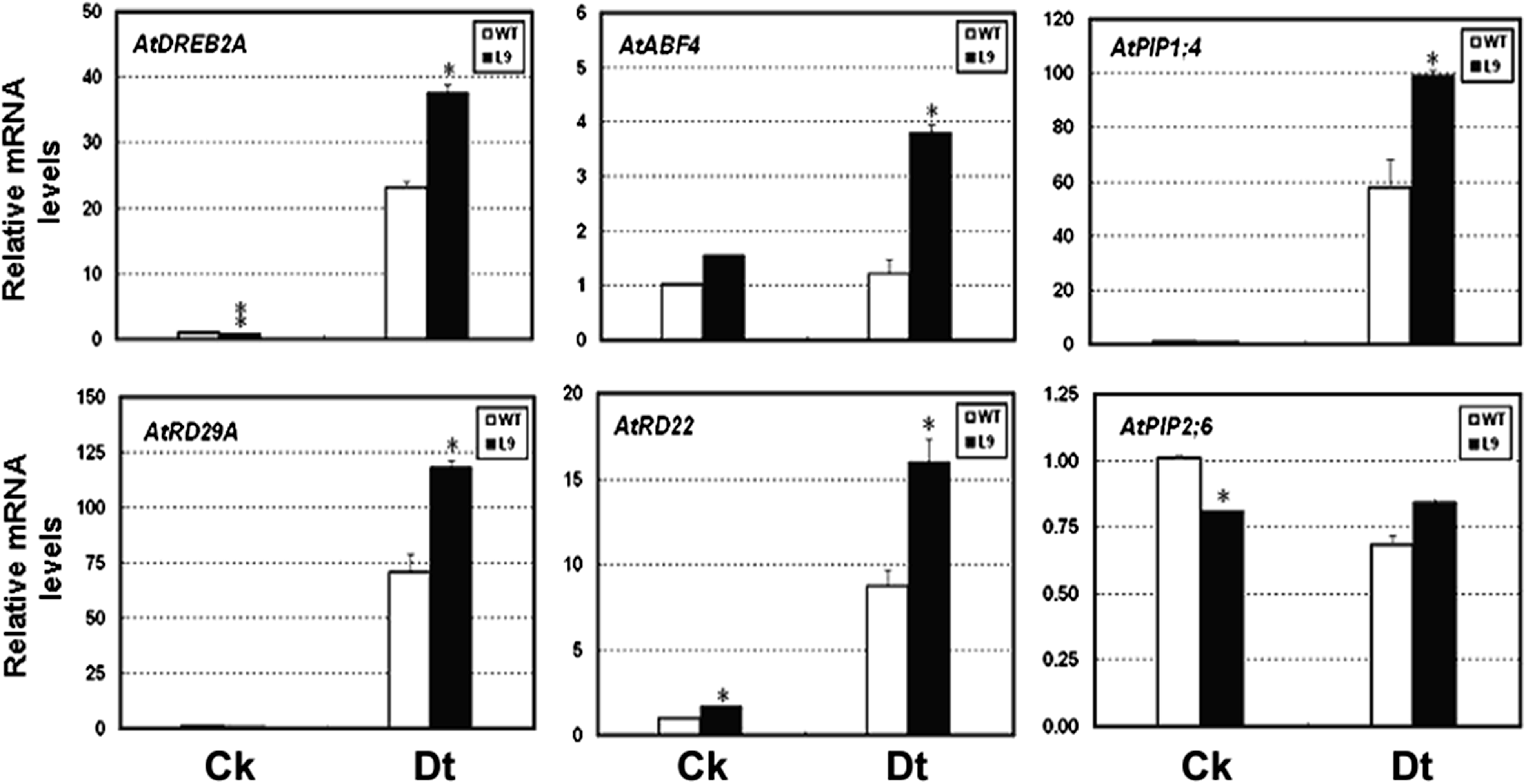

Supplement: Supplementary file 15 — Authors’ original file for figure 8 [file 12284_2012_34_MOESM15_ESM.tiff]
