# Supplementary material for: Comprehensive analysis of differentially expressed rice actin depolymerizing factor gene family and heterologous overexpression of OsADF3 confers Arabidopsis Thaliana drought tolerance
Source: Rice (N Y). 2012 Nov 27;5:33. doi: 10.1186/1939-8433-5-33 (PMC4883719; doi:10.1186/1939-8433-5-33)
Supplement: Supplementary file 7 — Additional file 7:Table S1. Gene-specific primer pairs used for RT-PCR (right) or real-time PCR (left) analysis of mRNA expression of rice actin depolymerizing factor (OsADF) in rice and in Arabidopsis. (PDF 39 KB) [file 12284_2012_34_MOESM7_ESM.pdf]

Additional file 2

Supplementary Table S1. Gene-specific primer pairs used for RT-PCR (right) or real-time PCR (left) analysis of mRNA expression of rice actin depolymerizing factor (*OsADF*) in rice and in *Arabidopsis*.

Gene name	Primer name	Sequence (5'- 3')	Amplicon (bp)
OsADF1	**OsADF1-F**	AAACACAGGCCGGTTACTGC	273
	**OsADF1-R**	ACCAGCATGGTGGAAGGACT	
OsADF2	**OsADF2-F**	CGATCACCTGCGAATCTCAA	235
	**OsADF2-R**	GGGAGAGGGTGTTTCATACG	
OsADF3	**OsADF3-F**	AGGACCACACCTCCTAATTAATCAC	162
	**OsADF3-R**	CGTCTCTTTATTATTCGCGTAATGT	
OsADF4	**OsADF4-F**	TGAATGGCATTCAGTTGGAA	226
	**OsADF4-R**	CGAAATACAGGATAGAGCCA	
OsADF5	**OsADF5-F**	GGCTTGCGGCGCTGTACTTA	250
	**OsADF5-R**	GTCACCGACATGGGACGTTT	
OsADF6	**OsADF6-F**	ATTAACCAAGATGTTCCCAA	230
	**OsADF6-R**	GATCATCTAGTTTACAGGAGTAC	
OsADF7	**OsADF7-F**	ATTTATTTAGCTTGACTGTCTGCC	251
	**OsADF7-R**	-AGTCGAATATGGCGTATCTG	
OsADF8	**OsADF8-F**	ATGGTGGGTGGCGGCTCGCC	200
	**OsADF8-R**	ACGGCGTCGGCCGGAAGCGA	
OsADF9	**OsADF9-F**	TCATATCATTTCATCATCCAACACC	362
	**OsADF9-R**	GCCAACAAATTGCATCAATACAACT	
OsADF10	**OsADF10-F**	AGAGATTGACTTCGATAGTTTG	206
	**OsADF10-R**	CCATATCATATTAGAGGGAA	
OsADF11	**OsADF11-F**	TCAATCAATCAATCACTAGG	156
	**OsADF11-R**	GCACAGTTGTACTTCAACAG	
OsUBI	**OsUBI-F**	CGCAAGTACAACCAGGACAA	101
	**OsUBI-R**	TGGTTGCTGTGACCACACTT	
AtUBC10	**AtUBC10-F**	TGGATATGGCGTCGAAGC	400
AT5G53300	**AtUBC10-R**	GTGGGATTTTCCATTTAGCC	

Gene name	Primer name	Sequence (5'- 3')	Amplicon (bp)
AtUBC10	**AtUBC10-Q-F**	CATCTTTGTGAAAACTAGAATCTGATCA	83
AT5G53300	**AtUBC10-Q-R**	TCACACAACGAAAACATCAAATCAT	
AtABF4	**AtABF4-Q-F**	CATTTTGTGCCCTGACCTTTG	87
AT3G19290	**AtABF4-Q-R**	TTAGAAACCCTAACAACCAGAAAGC	
AtRD22	**AtRD22-Q-F**	CTTCCTCCCGGAGACTCATG	75
AT5G25610	**AtRD22-Q-R**	TACATAACCCACAATAAGATAGAAAACAGATC	
AtDREB2A	**AtDREB2A-Q-F**	TGTTGATTCTCTATCCGAGTTTTAGTG	148
AT5G05410	**AtDREB2A-Q-R**	GAACAACTTAAGAGAAGCAGACTAACAAA	
AtRD29A	**AtRD29A-Q-F**	AAGGAGCTTTAAGAATATGAGAACTGAGA	78
AT5G52310	**AtRD29A-Q-R**	CATCAAAGACGTCAAACAAAACACA	
AtPIP1;4	**AtPIP1;4-Q-F**	GGCAAAGGGTCTCTGAAGTCTAAGG	129
At4g00430	**AtPIP1;4-Q-R**	AACCAACCCGAGAACTTGATGTTG	
AtPIP2;6	**AtPIP2;6-Q-F**	TTTCGAACTAGCGAAGAGGTGAAG	132
At2g39010	**AtPIP2;6-Q-R**	AGACACAGTAAATGTCACTCACCG	

(F: Forward primer; R: Reverse primer)

(Supplementary Table S1, Huang *et al.*, 2012)
